# Protein import into peroxisomes occurs through a nuclear pore-like phase

**DOI:** 10.1126/science.adf3971

**Published:** 2022-12-16

**Authors:** Yuan Gao, Michael L. Skowyra, Peiqiang Feng, Tom A. Rapoport

**Affiliations:** 1 Department of Cell Biology, Harvard Medical School, Boston, MA, 02115, USA.; 2 Howard Hughes Medical Institute, Harvard Medical School, Boston, MA, 02115, USA.

## Abstract

Peroxisomes are ubiquitous organelles whose dysfunction causes fatal human diseases. Most peroxisomal proteins are imported from the cytosol in a folded state by the soluble receptor PEX5. How folded cargo crosses the membrane is unknown. Here, we show that peroxisomal import is similar to nuclear transport. The peroxisomal membrane protein PEX13 contains a conserved tyrosine (Y) – and glycine (G)-rich YG domain, which forms a selective phase resembling that formed by FG repeats within nuclear pores. PEX13 resides in the membrane in two orientations that oligomerize and suspend the YG meshwork within the lipid bilayer. Purified YG domains form hydrogels into which PEX5 selectively partitions, using conserved aromatic amino acid motifs, bringing cargo along. The YG meshwork thus forms an aqueous conduit through which PEX5 delivers folded proteins into peroxisomes.

## ONE-SENTENCE SUMMARY

A tyrosine/glycine-rich YG domain of PEX13 forms a meshwork in the peroxisomal membrane through which proteins are imported.

Peroxisomes are organelles enclosed by a single membrane and are found in most eukaryotic cells (1). They provide vital functions including fatty acid oxidation (2) and detoxification of reactive oxygen species (3). Peroxisomes are essential for human health: various debilitating and often fatal disorders – notably the Zellweger spectrum – arise from defective import of enzymes into the peroxisomal lumen, otherwise known as the matrix (4).

Matrix proteins are made in the cytosol and then imported into peroxisomes. Most of them contain a type 1 peroxisome targeting signal (PTS1) at their C terminus, which comprises the amino acid sequence Ser-Lys-Leu (SKL) or variants of it (5). The PTS1 is recognized in the cytosol by the soluble receptor PEX5 through the receptor’s tetratricopeptide repeat (TPR) domain (6). PEX5 also contains a flexible N-terminal region that includes several aromatic motifs conforming to the amino acid sequence WxxxF/Y (where “x” denotes any residue) (7). Some matrix proteins contain an alternative N-terminal signal called PTS2, whose recognition requires the adapter PEX7 (8). In humans, PEX7 binds a short motif in PEX5, whereas in many fungi PEX7 associates with PEX5 paralogs that lack a TPR domain but retain the unstructured N-terminal region with its characteristic WxxxF/Y motifs (9).

PEX5 is recruited to peroxisomes by the membrane proteins PEX13 and PEX14 (and PEX17 in yeast) (7). Recruitment requires the receptor’s WxxxF/Y motifs (10), and is followed by translocation of the cargo-bound receptor completely into the matrix (10–12). To return to the cytosol, PEX5 is ubiquitinated by the PEX2-PEX10-PEX12 ubiquitin ligase complex (13), and pulled out through a pore in the ligase complex (14) by a hexameric ATPase consisting of alternating copies of PEX1 and PEX6 (15). Deubiquitination in the cytosol resets PEX5 for a new import cycle (16–17).

How PEX5 crosses the peroxisomal membrane to deliver cargo into the lumen has been a longstanding question. Particularly mysterious is the receptor’s ability to import folded or oligomeric proteins (18), or even gold beads (19). Thus, translocation into peroxisomes fundamentally differs from that into the endoplasmic reticulum (ER) or mitochondria, which can only import proteins in an unfolded conformation (20, 21). It is also puzzling that translocation across the peroxisomal membrane does not require nucleotide hydrolysis (22), even though import occurs against a concentration gradient of the cargo (23).

Translocation into peroxisomes is thought to be mediated by PEX13 or PEX14. While PEX14 has historically been favored (24), the protein lacks obvious features that could form an aqueous conduit for moving hydrophilic proteins across the membrane (25). In addition, PEX14 may be dispensable for import in some organisms (26, 27). PEX13 in contrast is essential for import in all organisms that have been tested (28, 29). Curiously, PEX13 contains an unstructured N-terminal region of unknown function that is required for import (30) and is enriched in the amino acids tyrosine and glycine (31). We noted that this tyrosine/glycine-rich YG domain resembles the phenylalanine/glycine-rich FG domains of nucleoporins, which reside within the nuclear pore and form a meshwork that restricts entry of large molecules into the nucleus (32). This meshwork is locally broken by nuclear transport receptors (NTRs), allowing them to rapidly diffuse through nuclear pores along with bound cargo (33).

Here, we show that the YG domain of PEX13 forms a similarly selective phase on peroxisomes. Our results lead to a model whereby the YG phase is locally disrupted by aromatic residues in the receptor’s WxxxF/Y motifs, allowing PEX5 to diffuse across the membrane into the matrix and carry cargo along. This mechanism explains how folded and oligomeric proteins are imported into peroxisomes.

## The YG domain of PEX13 is conserved and essential for peroxisomal import

**Fig. 1.**
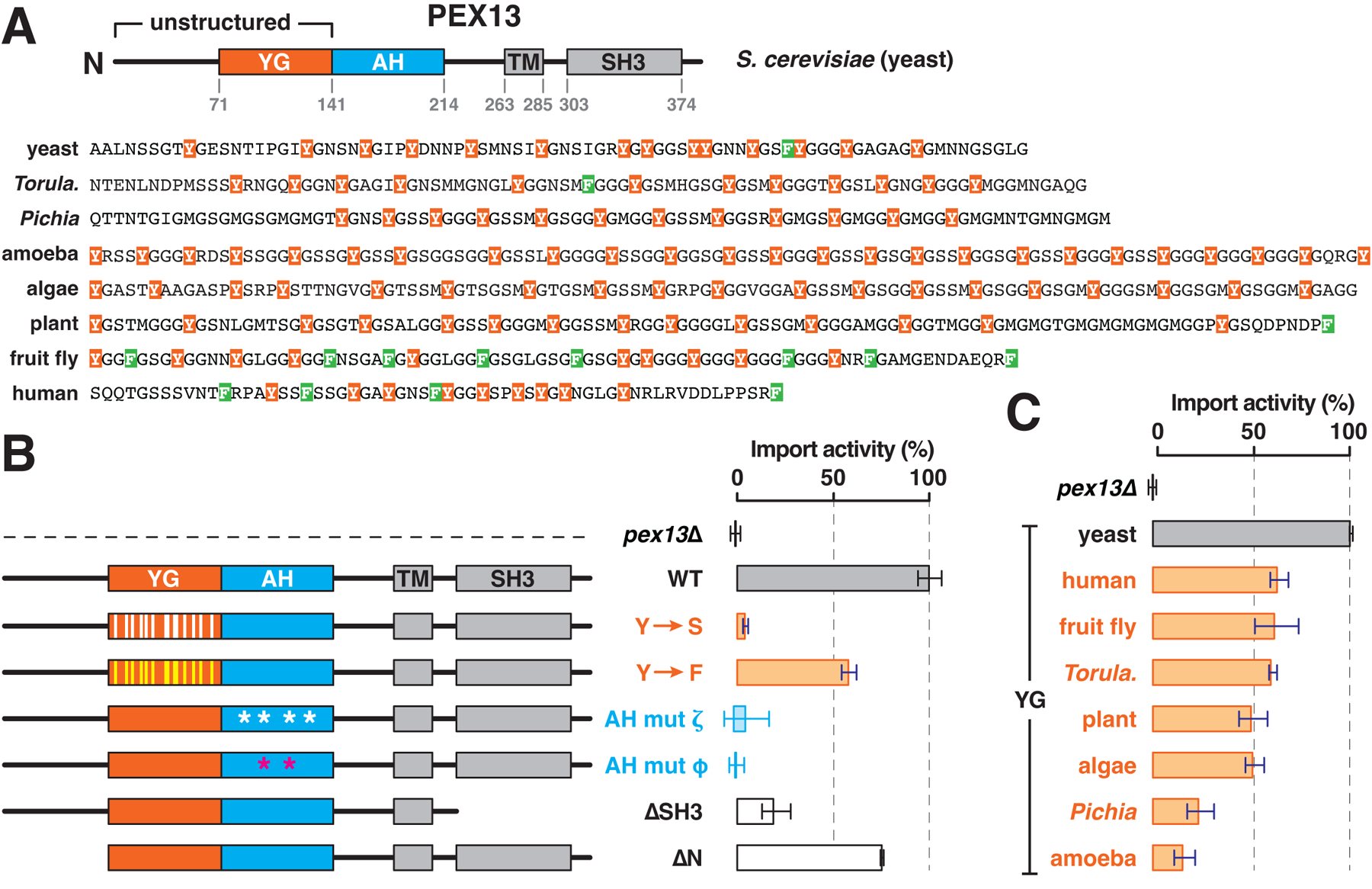
The YG domain of PEX13 is required for peroxisomal protein import. (**A**) The domain organization of PEX13 from *S. cerevisiae* (yeast) is shown at the top. Numbers denote amino acid coordinates. Sequences of the YG domain in PEX13 homologs from organisms representing the indicated eukaryotic clades (*Torula., Torulaspora*) are shown at the bottom. (**B**) Peroxisomal protein import activity (mean ± SE of three experiments) in yeast cells expressing the indicated PEX13 mutants compared with wild-type (WT) cells and a PEX13 knockout strain (pex13D). Y→S and Y→F denote conversion of all tyrosines (positions indicated by vertical bars) in the YG domain into serines or phenylalanines, respectively. AH mut ζ and φ denote conversion of four hydrophilic residues in the AH into alanine or two hydrophobic residues into glutamate, respectively. (**C**) Import activity in yeast cells expressing PEX13 chimeras with YG domains from the indicated organisms. Single-letter abbreviations for the amino acid residues are as follows: A, Ala; C, Cys; D, Asp; E, Glu; F, Phe; G, Gly; H, His; I, Ile; K, Lys; L, Leu; M, Met; N, Asn; P, Pro; Q, Gln; R, Arg; S, Ser; T, Thr; V, Val; W, Trp; and Y, Tyr.

The YG domain is found in PEX13 homologs from species representative of all eukaryotic clades (Fig. 1A). The domain is characterized by a preponderance of aromatic amino acids, predominantly tyrosine, but phenylalanines also occur in some organisms (Fig. 1A and fig. S1A). The number and position of the aromatic residues vary (Fig. 1A). The aromatic residues are separated by short linkers of approximately 4 amino acids (fig. S1B), which are enriched in small residues, notably glycine and serine, and lack charged residues (Fig. 1A and fig. S1A). These properties are generally similar to those of cohesive nucleoporin FG-repeat domains (fig. S1C), except that linkers between FG repeats are longer (fig. S1D).

The YG domain is followed by a long amphipathic helix (AH) (Fig. 1A and fig. S2), which has clearly defined hydrophobic and hydrophilic surfaces (fig. S3). Whether the AH consists of a straight helical segment, or has a kink in the middle, is unclear (fig. S3). Downstream of the AH, most PEX13 homologs contain a canonical TM and an SH3 domain that binds PEX5 (28) (Fig. 1A and fig. S2), although these are absent in some plants (34). Thus, only the YG domain and the AH are strictly conserved.

The YG domain is necessary for peroxisomal import, which we measured in the yeast *Saccharomyces cerevisiae* using an engineered pathway that generates a pigment when import is impaired (35). When all 14 tyrosines in the YG domain were mutated to serines, import was abolished (Fig. 1B), wheras converting all of the tyrosines into phenylalanines caused only a modest defect (Fig. 1B). Thus, the residues’ aromaticity is critical. A minimum of 11 tyrosines seems to be required in yeast (fig. S4A), but their position can be varied. The YG domain could also be replaced by the analogous domain from other organisms (Fig. 1C), albeit with variable efficiency, indicating that the domain’s function is conserved despite its sequence diversity. Taken together, these data demonstrate that the YG domain is a universal and essential feature of PEX13.

Import was also abolished by point mutations in either the hydrophilic face (mut ζ) or the hydrophobic face (mut φ) of the AH (Fig. 1B), revealing that the AH is likewise essential. While loss of the SH3 domain (ΔSH3) reduced import (Fig. 1B) as reported previously (36), deleting the flexible region that precedes the YG domain (ΔN) had no effect (Fig. 1B), indicating that this region is dispensable. We confirmed that all mutants were similarly expressed (figs. S4A–C).

## YG domains of multiple PEX13 molecules interact on peroxisomes

**Fig. 2.**
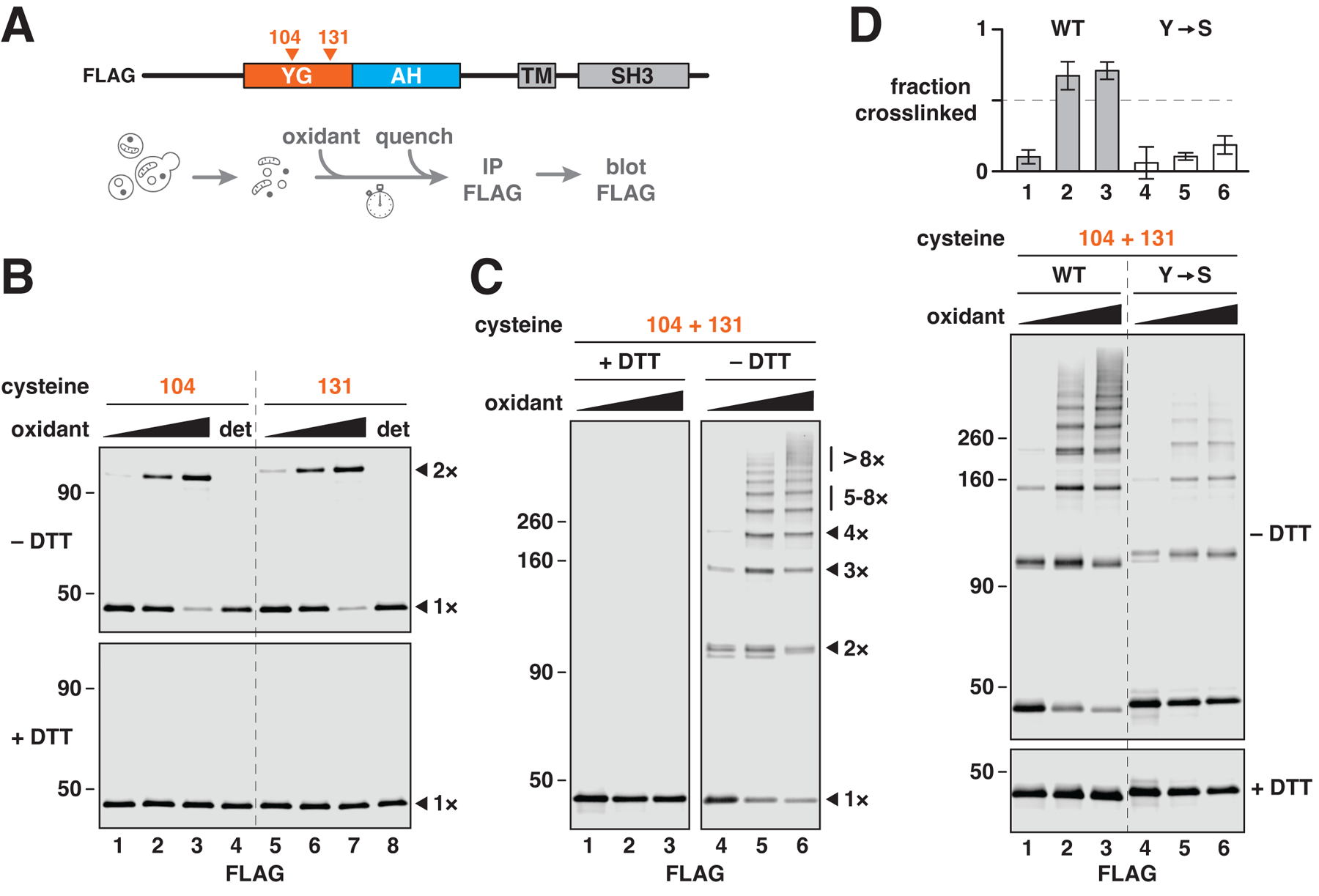
The YG domain of PEX13 forms oligomers in the peroxisomal membrane. (**A**) Scheme for the experiments shown in (B) to (D). To test whether YG domains associate with one another, cysteines were introduced at the indicated positions in the YG domain of FLAG-tagged yeast PEX13 (which lacks native cysteines), and the resulting constructs were expressed in yeast. Intact membranes from the corresponding strains were treated with Aldrithiol-4 (oxidant), unreacted cysteines were then quenched with NEM, and the proteins were immunoprecipitated (IP) and analyzed by immunoblotting (blot). (**B**) As described in (A), using PEX13 proteins containing individual cysteines at the indicated positions. Reactions were performed in the presence of 0, 50, or 200 μM oxidant with or without detergent (det) and resolved by nonreducing (–DTT) or reducing (+DTT) SDS–polyacrylamide gel electrophoresis (SDS-PAGE) before immunoblotting. Monomers (1×) and disulfide-linked dimers (2×) are indicated on the right. Numbers along the left side specify relative molecular weights (in kD). (**C**) Same as in (B), but with PEX13 containing two cysteines. The number of disulfide-linked molecules is indicated on the right (1× to 8×). (**D**) Same as in (C), but comparing PEX13 with tyrosines (WT) or serines (Y→S) in the YG domain. Cross-linking efficiency is plotted at the top (mean ± SE of three experiments).

To determine whether YG domains form a nuclear pore-like phase on peroxisomes, we first tested whether these domains associate with one another in the peroxisomal membrane. Individual cysteines were introduced at either of two positions (residues 104 or 131) in the YG domain of FLAG-tagged yeast PEX13 (which lacks natural cysteines) (Fig. 2A), without affecting import activity (fig. S5A). Formation of disulfide-linked dimers was then assessed using an oxidizing agent (Fig. 2A). Both positions indeed yielded efficient dimerization (>70 %) (Fig. 2B, upper panel). Dimers were disulfide-linked because the corresponding bands disappeared upon reduction with dithiothreitol (DTT) (Fig. 2B, lower panel). Dimerization was also sensitive to detergent (Fig. 2B, lanes 4 and 8), suggesting that the interaction requires an intact membrane. These results thus show that the YG domains of individual PEX13 molecules interact in the peroxisomal membrane.

The interaction between YG domains is in fact multivalent and does not involve just two molecules. When two cysteines were introduced into the YG domain (at positions 104 and 131), a ladder of disulfide-linked bands was observed (Fig. 2C, right panel). Some crosslinking occurred even without oxidant (lane 4), suggesting that the interaction is highly favored. The largest crosslinked species reveal that more than eight PEX13 molecules interact through their YG domains. Crosslinking was greatly reduced after converting all tyrosines in the YG domain into serines (Fig. 2D, compare lanes 3 and 6 and corresponding quantification). Crosslinking was unaffected by the absence of other import components including PEX2, PEX5, PEX14, or PEX17 (fig. S4D), suggesting that PEX13 forms oligomers by itself. This conclusion agrees with previous studies showing that PEX13 forms oligomers independently of other import components (37, 38). In summary, our analysis demonstrates that multiple PEX13 molecules associate with one another on peroxisomes through their YG domains, analogously to how nucleoporin FG domains interact within the nuclear pore.

## The YG domain is inaccessible to large molecules

**Fig. 3.**
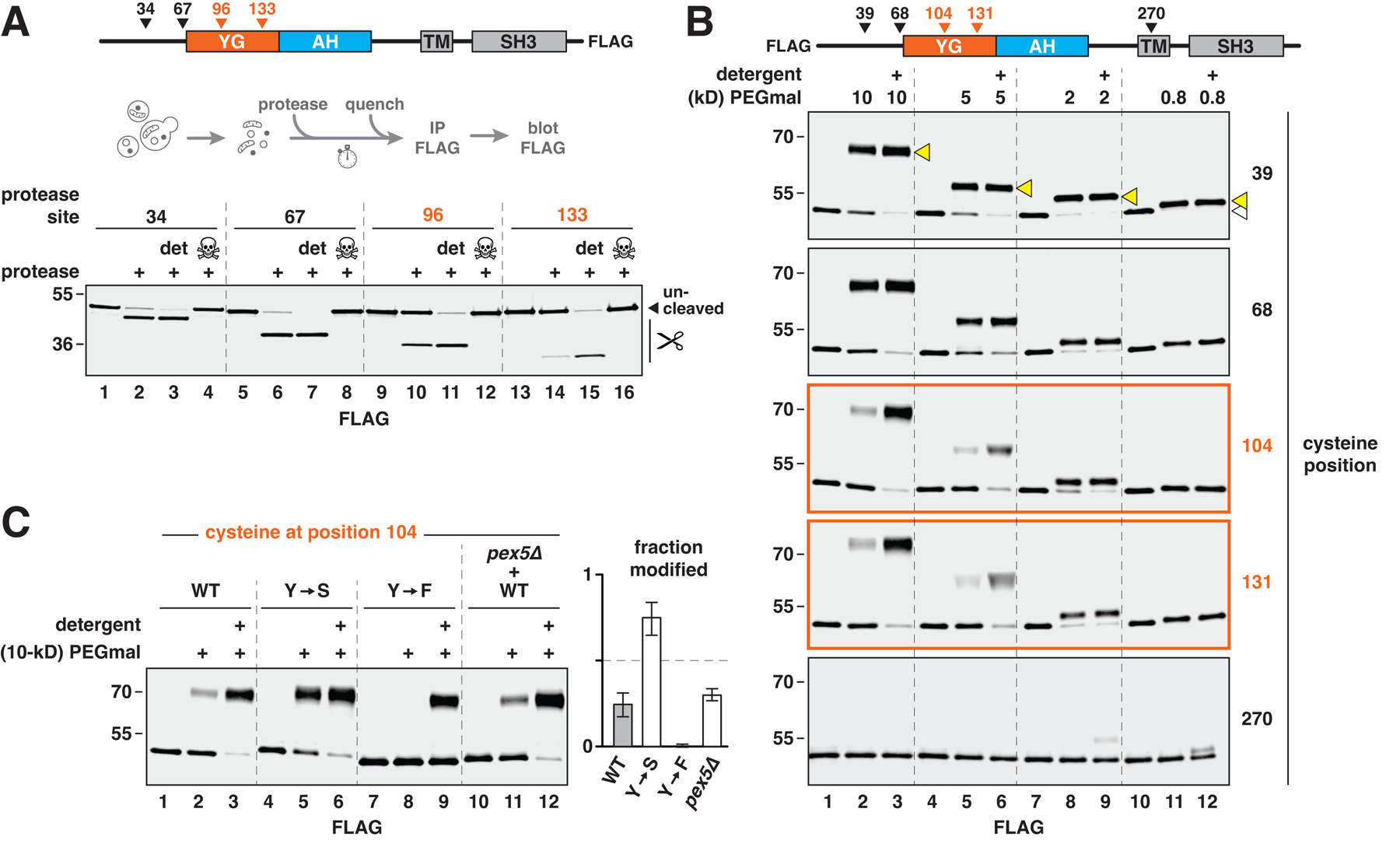
The YG domain is poorly accessible to large molecules. (**A**) To test accessibility of the YG domain to proteins, a 3C protease–cleavage site was introduced into FLAG-tagged yeast PEX13 at the indicated positions, and the resulting constructs were expressed in yeast. Intact membranes from each strain were first treated with the protease with or without detergent (det) and then quenched with NEM to inactivate the protease. Where indicated (skull and crossbones), NEM was added before the protease. Scissors designate the cleaved forms. (**B**) Same as in (A), except that accessibility of the YG domain to differently sized molecules was assessed by introducing individual cysteines into FLAG-tagged PEX13 at the positions shown. Membranes from the corresponding strains were treated with different sizes (in kD) of cysteine-reactive PEGmal and then quenched with excess cysteine. Covalent modification of the proteins was visualized by immunoblotting. Modified and unmodified forms of the protein are designated by yellow and white triangles, respectively, in the top blot. (**C**) Same as in (B), but with PEX13 containing a cysteine at position 104 and tyrosines in the YG domain (WT), or tyrosines mutated to serines (Y→S) or phenylalanines (Y→F). Where indicated, membranes were isolated from a strain lacking PEX5 (*pex5*Δ). Modification was performed with 10-kD PEGmal. Modification efficiency is plotted on the right (mean ± SE of four experiments).

We next asked whether the interaction between YG domains restricts access to soluble material, like the FG meshwork inside nuclear pores that excludes large proteins and other molecules. A 3C protease-cleavage site was inserted at different positions in the YG domain of FLAG-tagged PEX13, or outside this domain near the N terminus (Fig. 3A, scheme). The resulting constructs replaced endogenous PEX13 in yeast and were fully active (fig. S5B). When membranes isolated from the corresponding strains were treated with the protease, the constructs with N-terminal cleavage sites were readily digested (Fig. 3A, lanes 2 and 6), consistent with the reported cytosolic accessibility of the N terminus (39). In contrast, sites in the YG domain were much more resistant to cleavage (lanes 10 and 14) unless detergent was added (lanes 11 and 15). No cleavage occurred at any site when the protease was pre-inactivated by *N*-ethylmaleimide (NEM; lanes marked by crossbones). These data thus show that the YG domain is not easily accessed by the 30-kD 3C protease.

To confirm this result, we probed the accessibility of the YG domain to cysteine-reactive polyethylene glycol (PEGmal). Individual cysteines were introduced throughout FLAG-tagged PEX13 in yeast (Fig. 3B, scheme), and membranes from the corresponding strains were treated with different sizes of PEGmal. Cysteines near the N terminus were readily modified by all sizes of PEGmal (Fig. 3B, upper two blots) as expected. In contrast, cysteines in the YG domain were barely modified by the two largest PEGmal reagents (5 and 10 kD) unless detergent was included (lanes 1–6 in the two middle blots). This resistance to modification depended on size, because the two smallest forms of PEGmal (0.8 and 2 kD) efficiently modified either cysteine (lanes 7–12 in the two middle blots). As a control, we tested the accessibility of a cysteine in the TM, which was not modified by PEGmal of any size (bottom blot).

Converting all tyrosines in the YG domain to serines (Y→S) increased accessibility to 10-kD PEGmal (Fig. 3C), consistent with this mutant’s reduced self-association (Fig. 2D). Converting all tyrosines to phenylalanines (Y→F) instead improved the resistance to modification (Fig. 3C), again highlighting the importance of the aromatic residues in cementing the interaction between PEX13 molecules. The absence of PEX5 (*pex5Δ*) had no effect on accessibility (Fig. 3C), suggesting that active import may not be necessary for the YG domain’s exclusion properties. Taken together, our results reveal that multiple YG domains form a meshwork in the peroxisomal membrane, which excludes large molecules but not small ones, similarly to the FG meshwork in nuclear pores.

To confirm this conclusion, we introduced single cysteines on either side of the transmembrane segment of the peroxisomal membrane protein PEX14 (which also lacks native cysteines), and examined their accessibility to different sizes of PEGmal as above (fig. S6A). The C terminus of PEX14 faces the cytosol (10, 39), and accordingly, a cysteine located near the C terminus (position 242) was readily modified by all sizes of PEGmal in the absence of detergent (fig. S6B, upper blot). In contrast, a cysteine located near the N terminus (position 65), which is oriented toward the lumen (10, 39), was modified only by the smallest PEGmal tested (fig. S6B, lower blot). In the presence of detergent, both cysteines were indiscriminately modified by all sizes of the reagent. These results thus confirm that the peroxisomal membrane is permeable to small molecules, as suggested before (35, 40), and are consistent with a dense meshwork of limited porosity residing in the membrane.

## PEX13 adopts two orientations in the peroxisomal membrane

**Fig. 4.**
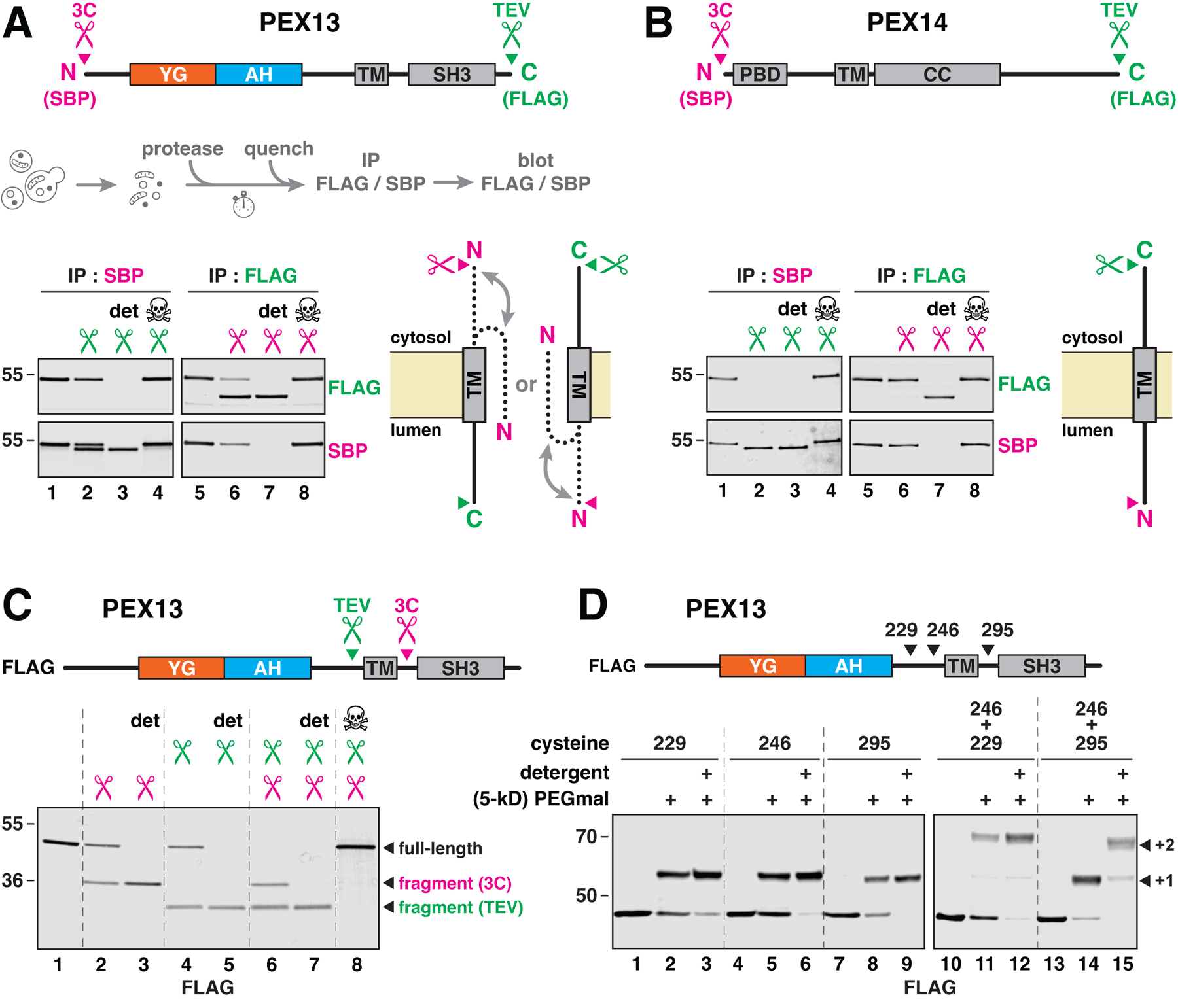
PEX13 adopts two transmembrane orientations. (**A**) Membrane topology determined by protease protection. The indicated protease-cleavage sites (scissors) and epitope tags were introduced into yeast PEX13. Membranes containing this protein were treated with protease with or without detergent (det), the reactions were quenched with NEM, and PEX13 was immunoprecipitated (IP); cleavage was visualized by immunoblotting (see scheme). Where indicated (skull and crossbones), NEM was added before the proteases. The position of the C terminus is deduced from cleavage with TEV protease and immunoprecipitation by the N-terminal SBP tag, and the position of the N terminus is deduced from cleavage with 3C protease and immunoprecipitation by the C-terminal FLAG tag. The two inferred orientations of PEX13 are depicted on the right; the N terminus can face either side, whereas the C terminus is fixed in one of two orientations. (**B**) Same as in (A), but for PEX14. PBD, PEX5- binding domain; TM, transmembrane segment; CC, coiled-coil oligomerization domain. (**C**) Same as in (A), but with protease sites flanking the TM and a FLAG tag as shown. (**D**) TM orientation determined by modification of flanking cysteines with membrane-impermeable PEGmal. One or two cysteines were introduced into FLAG-tagged PEX13, as indicated. Membranes were treated with 5-kD PEGmal and then quenched with excess cysteine; single (+1) or double (+2) modification was visualized by immunoblotting.

The YG meshwork must be suspended within the peroxisomal membrane in order to form a conduit for cargo. To determine how the YG meshwork is formed, we examined the membrane topology of PEX13. The N terminus of the protein was fused to a 3C protease-cleavage site preceded by an SBP tag, and the C terminus was fused to a TEV protease-cleavage site followed by a FLAG tag (Fig. 4A, upper scheme). The construct was expressed from the endogenous locus in yeast and supported peroxisomal protein import (fig. S5B).

We first ascertained the orientation of the C terminus. Membranes were treated with TEV protease and PEX13 was then immunoprecipitated by the N-terminal SBP tag (Fig. 4A). Interestingly, only half of the PEX13 population was cleaved (lane 2; quantification in fig. S7A). The resistant pool was protected by the membrane, as it could be cleaved in the presence of detergent (Fig. 4A, lane 3). No cleavage occurred when the protease was pre-inactivated by NEM (lane 4). These data thus suggest that PEX13 resides in the membrane in two orientations: one whose C terminus faces the cytosol, and another whose C terminus faces the lumen (Fig. 4A, bottom scheme). The interaction between YG domains in the membrane might thus be mediated by PEX13 molecules of both orientations (see below).

To infer the orientation of the N terminus, membranes were treated with 3C protease, and PEX13 was then immunoprecipitated by the C-terminal FLAG tag (Fig. 4A). In contrast to the C terminus, more than 50 % of the molecules had their N termini accessible to the protease (lane 6), and the exact proportion varied between experiments (fig. S7A). The remaining pool was again cleaved in detergent (Fig. 4A, lane 7). The N terminus might thus not be fixed in one specific orientation, but may instead be free to move between the cytosol and the lumen (Fig. 4A, bottom scheme). Notably, these results agree with the cytosolic accessibility of the N terminus reported above (Figs. 3A and B).

Controls were performed with PEX14 (Fig. 4B) and PEX17 (fig. S7B), which are conventional single-pass membrane proteins whose N termini face the lumen and C termini the cytosol (10, 25, 39). Using analogously tagged constructs that supported peroxisomal import (fig. S5B), we found that the C termini of both proteins were fully cleaved in the absence of detergent, whereas their N termini were completely protected unless detergent was included (Fig. 4B and fig. S7B). The topologies of PEX14 and PEX17 thus agree with previous reports.

The dual topology of PEX13 is supported by two observed orientations of the protein’s TM. We incorporated 3C and TEV protease-cleavage sites on either side of the TM, and added a FLAG tag to the N terminus for immunodetection (Fig. 4C, scheme). The resulting construct allowed peroxisomal import (fig. S5B). Again, half of the molecules were accessible to either protease in the absence of detergent (Fig. 4C, lanes 2 and 4), whereas the entire population was cleaved when detergent was included (lanes 3 and 5). Notably, addition of both proteases produced both cleavage products (lanes 6–8), consistent with two orientations of the protein in the membrane.

We confirmed the dual topology of PEX13 by PEGmal accessibility. Single cysteines were introduced on either side of the TM (Fig. 4D, scheme) without affecting import (fig. S5A), and membranes from the corresponding strains were treated with 5-kD PEGmal. Regardless of whether the cysteine resided upstream (position 229 or 246) or downstream (position 295) of the TM, about half of the molecules showed the increase in molecular weight expected from cysteine modification (Fig. 4D, left blot). All molecules were modified in detergent. With two cysteines on the same side of the TM (positions 229 and 246), half of the PEX13 molecules were modified on both cysteines, whereas the other half were not modified at all (Fig. 4D, right blot), as expected. In contrast, with two cysteines on opposite sides (positions 246 and 295), essentially all PEX13 molecules were modified but only on one or the other cysteine. These results confirm that the TM of PEX13 spans the membrane in two possible orientations.

The dual topology does not require formation of the YG meshwork, as both orientations were retained after mutating all tyrosines in the YG domain into serines (fig. S8A). Both orientations were also retained in the absence of PEX2, PEX5, PEX8, PEX14, or PEX17 (fig. S8B), indicating that other import components are dispensable. In contrast, in the absence of PEX3, all PEX13 molecules became accessible to the proteases and the abundance of PEX13 was also reduced (fig. S8C). This observation agrees with previous reports of PEX13 being destabilized in the absence of PEX3 (41, 42) and with the proposed role of PEX3 in peroxisomal membrane protein insertion (43).

## The YG domain bridges both orientations of PEX13

**Figure 5.**
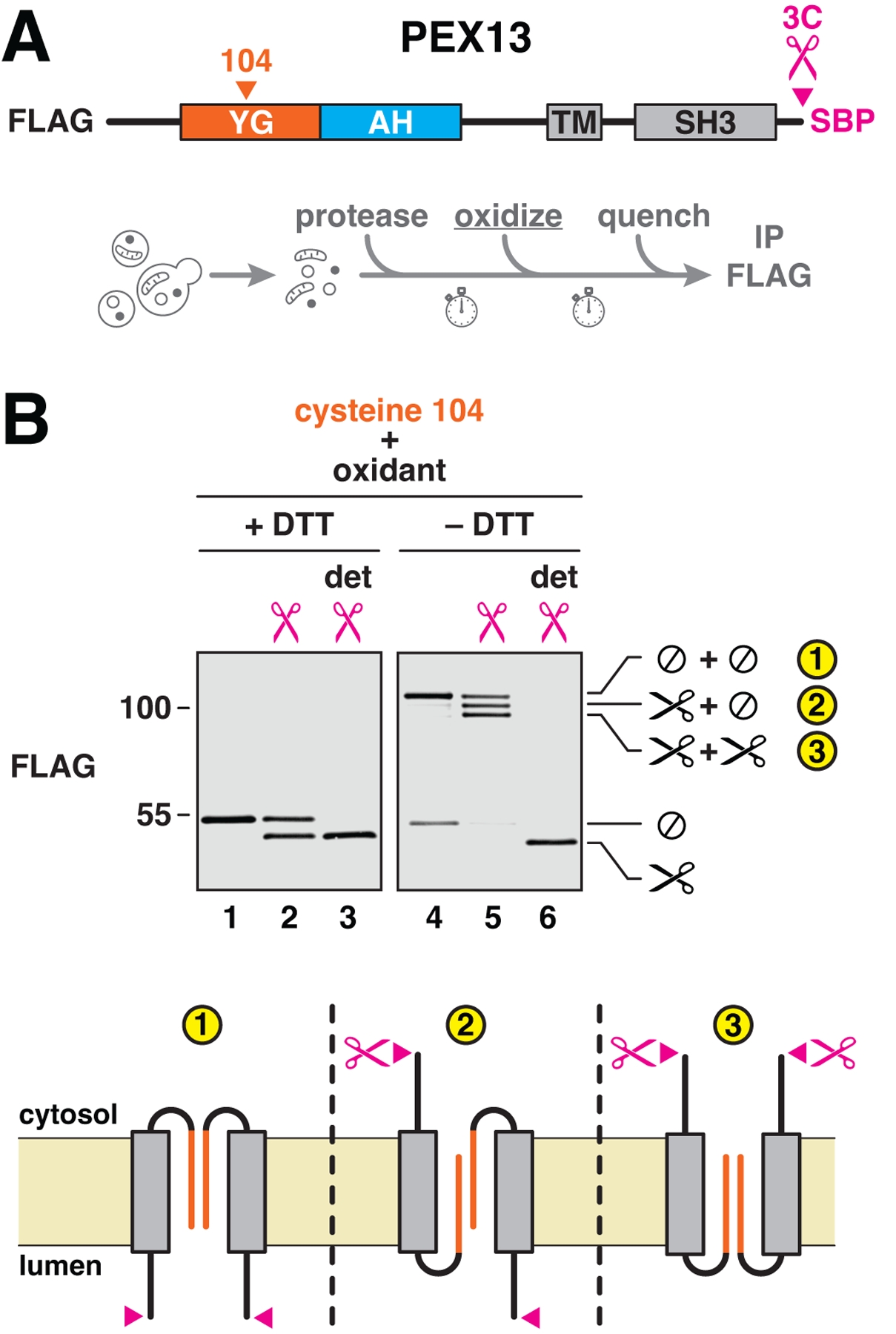
The two orientations of PEX13 are bridged by the YG domain. (**A**) To test whether the two orientations of PEX13 associate with each other, a single cysteine and a 3C protease–cleavage site were incorporated into FLAG-tagged yeast PEX13 as shown. An SBP tag was included at the C terminus to enhance the size shift after proteolytic cleavage. The resulting construct was integrated into yeast. Intact membranes from the corresponding strain were treated with the protease to reveal the protein’s two orientations, oxidized with Aldrithiol-4 to induce disulfide-bridge formation, and quenched to inactivate the protease and oxidant. (**B**) Disulfide-linked dimers were visualized by reducing (+DTT) or nonreducing (–DTT) SDS-PAGE and immunoblotting for the FLAG tag. Where indicated, detergent (det) was included during protease cleavage. Cleaved (scissors) and uncleaved (⊘) species are marked on the right. The topologies of the three observed dimers (numbered) are depicted below.

We next asked whether the two orientations of PEX13 associate with each other, allowing the YG domains of these molecules to interact within the membrane. We introduced a single cysteine into the YG domain and tested whether disulfide-linked dimers would form between the two orientations. To discern both orientations, the C terminus was fused to a 3C protease-cleavage site followed by an SBP tag (to enhance the size shift), and an N-terminal FLAG tag was added for immunodetection (Fig. 5A). Cleavage by the protease produced two bands of equal intensity in a reducing gel (Fig. 5B, left blot), corresponding to the two orientations of the C terminus. Under oxidizing conditions, however, three disulfide-linked bands were seen after cleavage (right blot), corresponding to PEX13 dimers in which the two molecules have the same or opposite topologies (Fig. 5B, scheme). These results establish that both orientations of PEX13 directly interact with each other through their YG domains, and thereby explain how the YG meshwork is localized to the plane of the membrane.

## YG domains form hydrogels

**Fig. 6.**
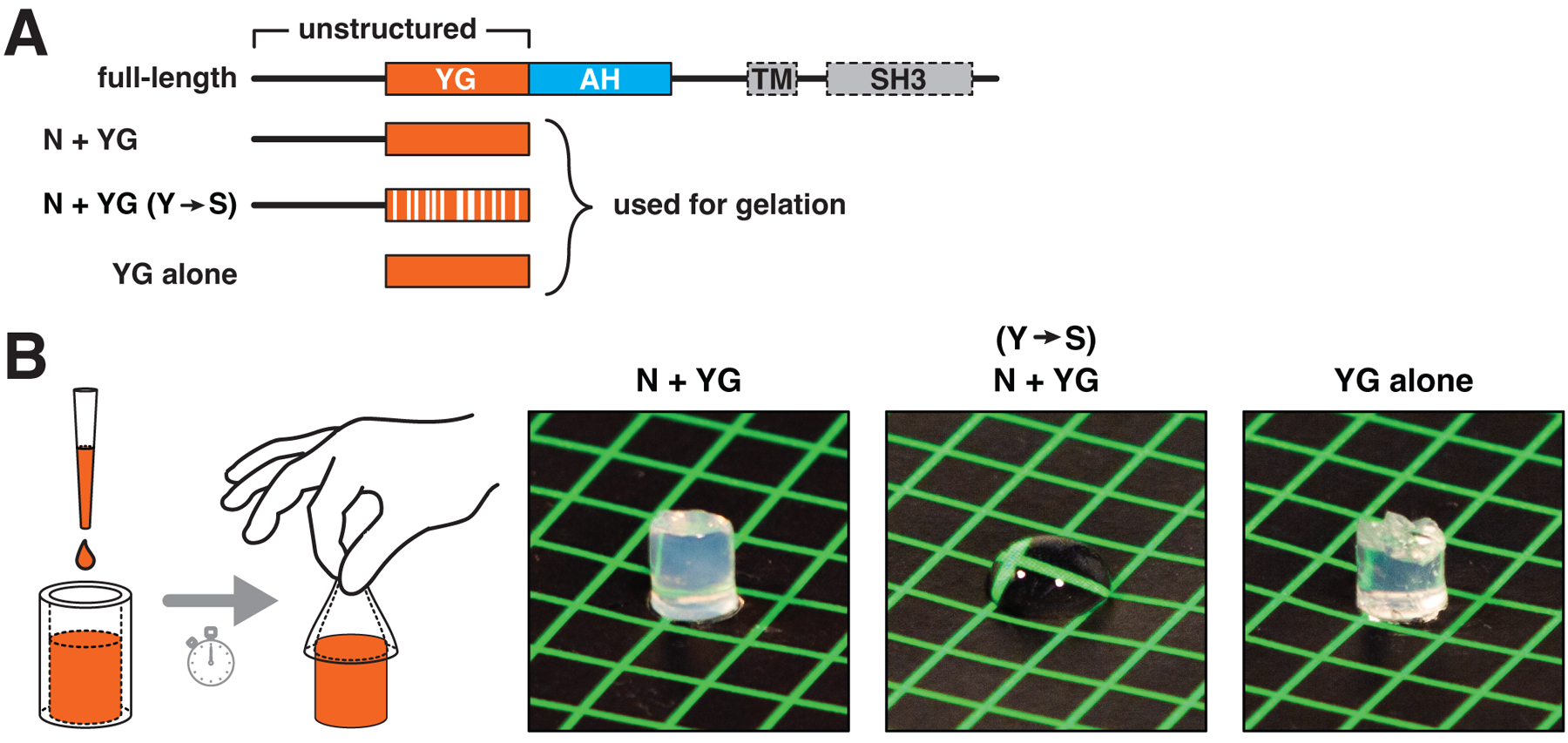
The YG domain of PEX13 forms hydrogels. (**A**) Scheme depicts the domain organization of *A. thaliana* PEX13, which lacks a TM and an SH3 domain (enclosed by dashed lines). The fragments indicated below were expressed in *Escherichia coli* and purified. Y→S denotes conversion of all tyrosines in the YG domain into serines. (**B**) Concentrated solutions (40 mg/ml) of the three purified fragments were pipetted into silicone tubing and allowed to gel and then squeezed out of the tubing onto a colored surface and photographed. If gelation had occurred, the solution retained the shape of the tubing; otherwise, the solution remained fluid. Note that the YG domain is necessary and sufficient for gelation.

Given that the nucleoporin FG meshwork can be reconstituted as a hydrogel (44) whose permeation properties mimic transport through the nuclear pore (45), we wondered whether the YG domain of PEX13 might behave similarly. After screening expression of YG domains from different organisms, we found that the entire unstructured N-terminal region (including the YG domain) from *Arabidopsis thaliana* PEX13 (Fig. 6A) expressed well in bacteria and could be purified under denaturing conditions using a His tag. Notably, the YG domain from this species could complement the yeast YG domain in peroxisomal import (Fig. 1C). After proteolytic removal of the tag (fig. S9) and concentration to 40 mg/ml (2 mM) in the presence of denaturant, the fragment indeed formed a rigid, translucent gel after several hours (Fig. 6B, left photograph). Higher concentrations could not be reached because the fragment gelled spontaneously, even when denaturant was included to retard gelation. A more dilute solution at 4 mg/ml (0.2 mM) also formed a hydrogel but after several days.

The YG domain is necessary and sufficient for gelation. When all tyrosines in the YG domain were mutated to serines (Y→S; Fig. 6A), a solution of the corresponding purified fragment never formed a gel and remained fluid (Fig. 6B, middle photograph). Furthermore, the isolated YG domain (without the upstream flexible segment; Fig. 6A) gelled as readily as the full-length fragment (Fig. 6B, right photograph). Interestingly, the gels liquified after heating to >50 °C, and re-solidified after cooling to room temperature; this cycle could be repeated many times. The purified protein thus behaves like gelatin, even though the amino acid compositions of the two proteins are very different. This reversible thermosensitivity differs from that of the FG domain of the nucleoporin NUP98, whose phase separation is more favorable at higher temperatures (46). The difference might be due to hydrogen bonds between tyrosine hydroxyl groups contributing to the cohesion of the YG hydrogel.

## PEX5 partitions into YG hydrogels

**Fig. 7.**
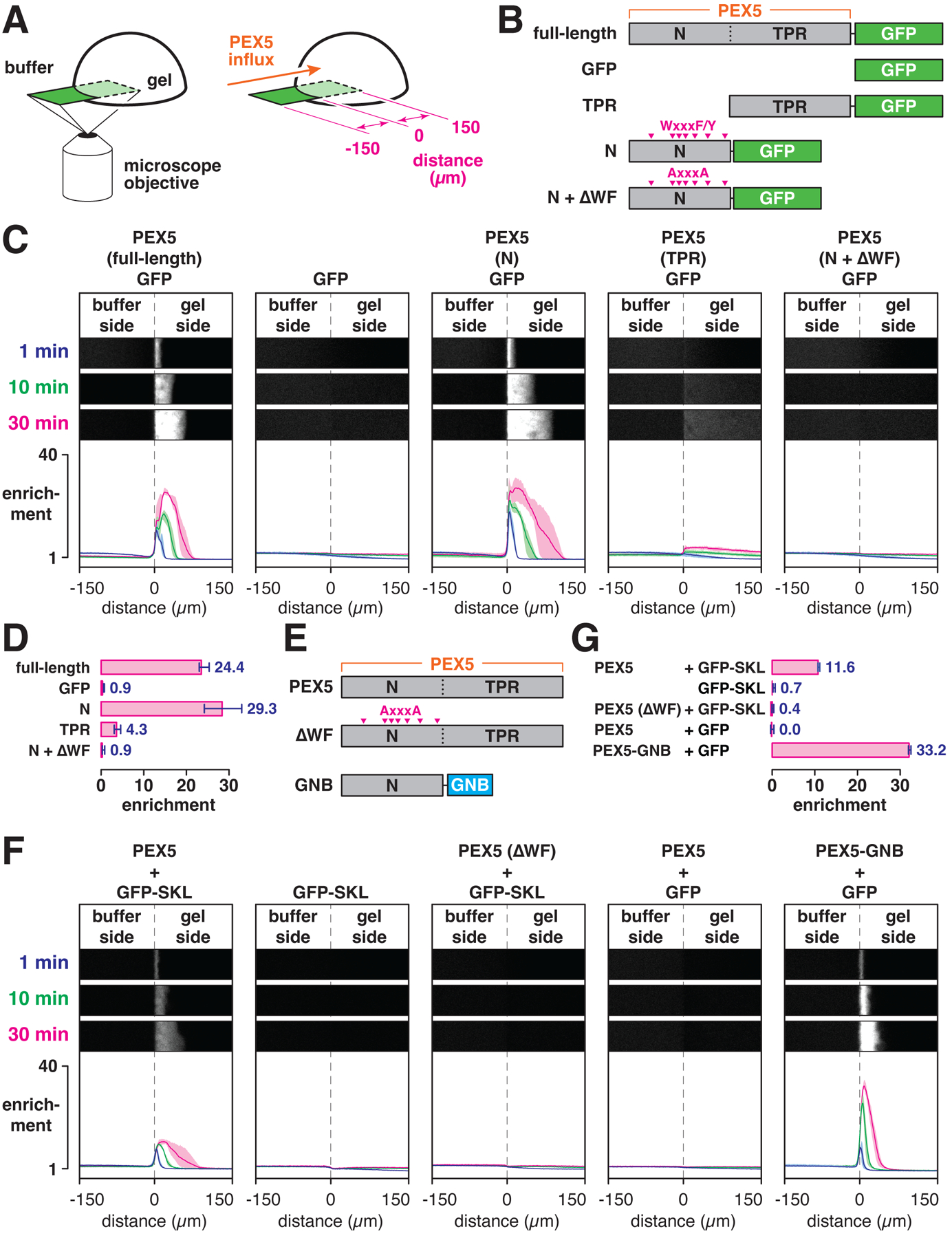
The import receptor PEX5 selectively enters YG hydrogels and brings cargo along. (**A**) YG-hydrogel droplets (40 mg/ml) were prepared in glass-bottomed dishes; permeation of the gels by fluorescently labeled PEX5 or other proteins was imaged by point-scanning confocal microscopy. (**B**) Scheme depicting the PEX5 fragments that were fused to GFP. PEX5’s N-terminal region contains several WXXXF/Y motifs (magenta arrows), which were mutated to AXXXA in the N + ΔWF mutant. (**C**) YG-hydrogel droplets were bathed in buffer containing the indicated GFP-fusion proteins (or GFP alone), and the interface between the buffer and gel was imaged over time. Shown are three selected time points; the fold enrichment of each protein, relative to buffer, across the imaged field is plotted below (mean ± the range of three experiments). (**D**) Mean enrichment (± the range of three experiments) of the indicated proteins inside the gel compared to buffer after 30 min of imaging as in (C). (**E**) Nonfluorescent PEX5 variants used for experiments with fluorescent cargo. GNB, anti-GFP nanobody. (**F**) Same as in (C) except with the indicated PEX5 variants, and GFP with or without the PEX5-binding SKL signal. (**G**) Mean enrichment (± the range of three experiments) of GFP or GFP-SKL after 30 min of imaging as in (F).

We next examined the permeation properties of the YG hydrogels. Small drops of the N-terminal fragment were gelled at 40 mg/ml in a glass-bottomed dish, and influx of fluorescently-labeled PEX5 or other proteins was imaged on a microscope (Fig. 7A). When buffer was added containing full-length PEX5 fused to GFP (PEX5-GFP, Fig. 7B), the fusion protein rapidly accumulated at the gel edge and then moved inward (Fig. 7C) at a rate of ~16 nm/sec (fig. S10). PEX5-GFP was depleted from the buffer just outside the gel, indicating that the rate of entry into the gel is limited only by diffusion. Fluorescence behind the front was essentially constant in the gel, indicating that PEX5-binding sites in this region became saturated. After 30 min of permeation, the concentration of PEX5-GFP inside the gel was ~20-fold greater than the protein’s concentration in the buffer (Fig. 7D). While GFP alone could also enter the gel, it showed no enrichment (Figs. 7C and D). Thus, PEX5 efficiently and specifically partitions into the YG hydrogel, analogously to how NTRs partition into hydrogels formed from nucleoporin FG domains.

Admission of PEX5 into YG hydrogels requires the receptor’s unstructured N terminus. When just the N-terminal region of PEX5 (including all WxxxF/Y motifs) was fused to GFP (Fig. 7B), the resulting fusion protein permeated the gel as rapidly as the full-length version (Fig. 7C) and became similarly enriched (Fig. 7D). In contrast, a fusion between the cargo-binding TPR domain and GFP (Fig. 7B) accumulated to a much lower extent (Figs. 7C and D). Entry into the gel was driven by the receptor’s WxxxF/Y motifs, since ablating all of the motifs (Fig. 7B) abolished the accumulation of the N-terminal fusion protein inside the gel (Figs. 7C and D).

PEX5 could also drag cargo into the YG hydrogels. GFP fused to a PTS1 import signal (GFP-SKL) showed no enrichment inside the gel on its own, but accumulated ~10-fold in the presence of full-length PEX5 (Figs. 7E–G). The lower partitioning coefficient likely reflects the modest affinity between the import signal and the receptor’s TPR domain (47). The permeation rate was comparable to that of the linear PEX5-GFP fusion (fig. S10). Partitioning of cargo required the receptor’s WxxxF/Y motifs, as full-length PEX5 lacking all of the motifs (Fig. 7E) was unable to drag GFP-SKL into the gel (Figs. 7F and G). While the TPR domain is necessary for the interaction with the import signal, the domain is dispensable for the actual partitioning. Indeed, GFP lacking the import signal was completely inert in the presence of full-length PEX5, but could be efficiently brought into the gel by a construct consisting of the receptor’s N-terminal region fused to a GFP nanobody (PEX5-GNB; Figs. 7E–F). In this case, GFP was enriched ~30-fold inside the gel (Fig. 7G), consistent with the higher affinity of the nanobody for the fluorescent protein (48). In summary, these results demonstrate that the YG domain of PEX13 forms hydrogels which selectively admit PEX5 and PEX5•cargo complexes.

## Discussion

**Fig. 8.**
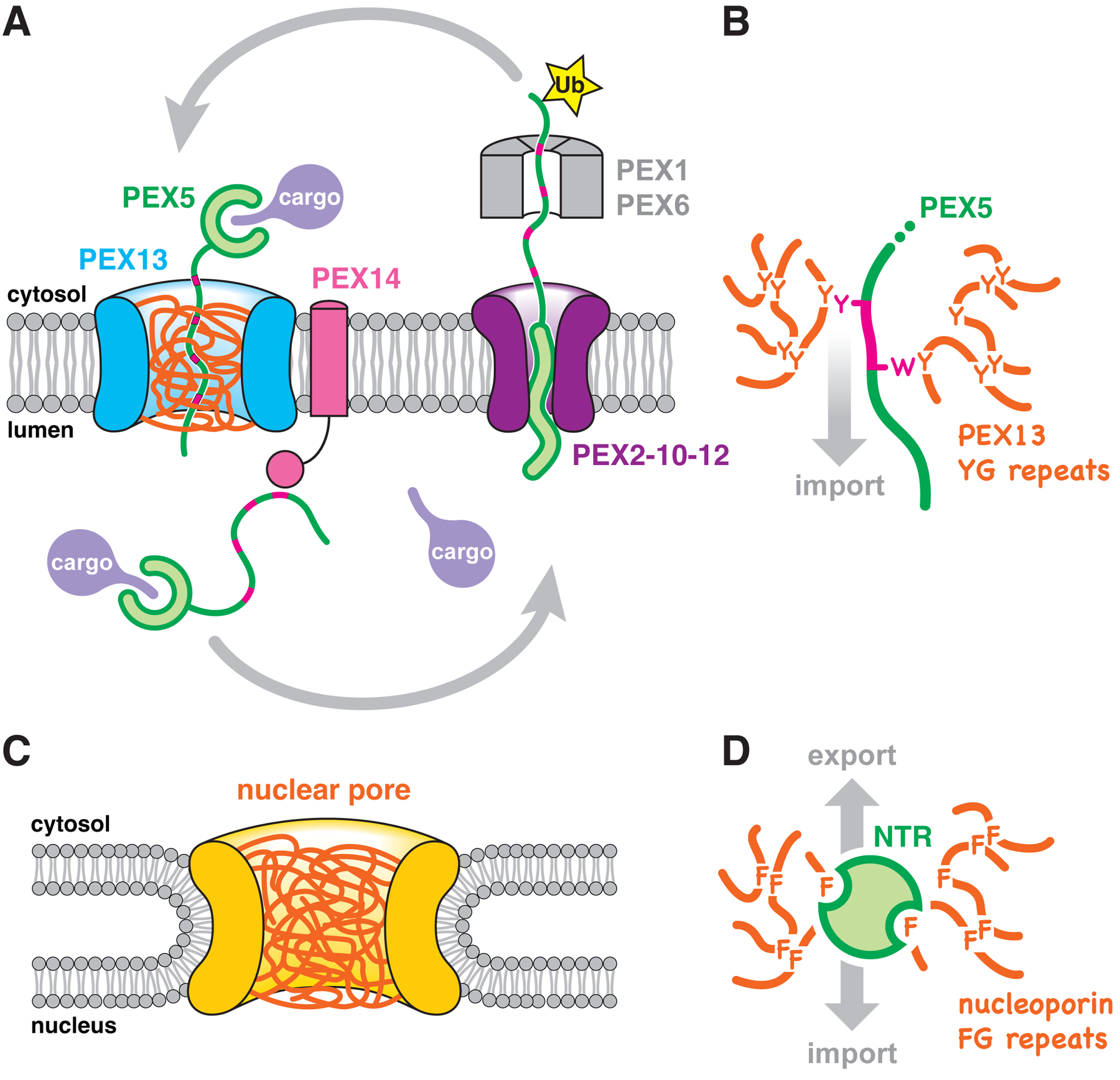
Model of peroxisomal matrix protein import. (**A**) PEX13 molecules form a conduit (blue) in the peroxisomal membrane filled with a meshwork (orange) of their YG domains. PEX5 crosses this barrier with bound cargo, using WXXXF/Y motifs (magenta) in its flexible N-terminal region. The interaction between the WXXXF/Y motifs and the lumenal domain of PEX14 retains the receptor inside the organelle. To return to the cytosol, PEX5 is monoubiquitinated (Ub) by the PEX2–10-12 ubiquitin ligase complex and pulled out by the PEX1-PEX6 ATPase. Unfolding of PEX5 during export enables cargo to be released in the lumen. After refolding in the cytosol, and having Ub removed by deubiquitinases, PEX5 can begin another import cycle. (**B**) PEX5 partitions into the YG meshwork as an extended polypeptide, whose WXXXF/Y motifs locally disrupt the cohesive tyrosine interactions that hold the meshwork together. (**C**) Diagram illustrating the scaffold (yellow) of the nuclear pore complex, filled with a meshwork of nucleoporin FG domains (orange). Note how the nuclear pore is suspended outside the bilayer instead of being embedded in it like the peroxisomal pore. (**D**) NTRs use hydrophobic pockets and patches in folded domains to partition into the FG meshwork and diffuse through the nuclear pore.

Our results with intact yeast membranes and synthetic hydrogels reveal the existence of a nuclear pore-like conduit on peroxisomes (Fig. 8A). This conduit consists of a dense meshwork – a selective phase – assembled from the YG domains of multiple PEX13 molecules that sit in the membrane in opposite orientations. Similarly to the meshwork formed by cohesive nucleoporin FG domains inside nuclear pores, the YG phase restricts passage of large soluble molecules. The YG phase can be selectively traversed by the import receptor PEX5, allowing bound cargo to move across the peroxisomal membrane. This mechanism thus allows folded and oligomeric proteins to be imported into peroxisomes, analogously to how folded cargo is moved into and out of the nucleus.

The use of tyrosines to form a selective phase is not unprecedented. For example, the protein FUS relies on tyrosine repeats to form hydrogels in vitro and to phase-separate into stress granules in vivo (49). Nucleoporin FG domains with tyrosines in place of phenylalanines also retain their ability to form hydrogels (44). Although these gels no longer accommodate NTRs, they do allow the entry of proteins engineered to bind the modified phase (50).

The permeation rate of PEX5 into the peroxisomal YG hydrogels (~16 nm/sec) implies that cargo would traverse the 4-nm peroxisomal membrane in under a second, which seems physiologically reasonable, although translocation rates in vivo are not known. PEX5 presumably enters the YG phase by locally dissolving the meshwork, relying predominantly on the WxxxF/Y motifs in its flexible N-terminal region (Fig. 8B). Entry of PEX5 into the YG phase might be regulated by cargo binding, as the association of the receptor with peroxisomes has been reported to rely on the presence of cargo (51). The interaction between PEX5 and the YG phase must be weak and transient, allowing the receptor to rapidly bind and dissociate to diffuse through the meshwork. The receptor’s TPR domain also has some affinity for the YG phase and would therefore enhance diffusion, which might be relevant for translocation of larger cargo. However, only the WxxxF/Y motifs are strictly conserved among peroxisomal import receptors (1), suggesting that they are sufficient to drive import both of PTS1 and PTS2 proteins.

The proposed mechanism implies that peroxisomal import receptors can diffuse through the YG phase in either direction, similarly to how NTRs move bidirectionally through the nuclear pore. Whereas the directionality of nuclear transport is enforced by the Ran•GTP/GDP gradient (GTP, guanosine triphosphate; GDP, guanosine diphosphate), peroxisomal import may be driven instead by the highly favorable interaction between the receptors’ WxxxF/Y motifs and the lumenal domain of the membrane protein PEX14 (52). This interaction might help pull PEX5 out of the YG phase on the lumenal side, and would also prevent retrograde diffusion of the receptor back into the cytosol (Fig. 8A). The interaction is likely potentiated by avidity, as import receptors frequently have several WxxxF/Y motifs (53), and PEX14 is known to form oligomers that associate with PEX13 (25, 54, 55). Sustained import would thus require unliganded PEX14, which could only be generated by continual retrieval of PEX5 from the lumen by ATP-dependent recycling (Fig. 8A). This model explains why import per se is independent of nucleotide hydrolysis and yet occurs against cargoes’ concentration gradient: the energy derives from PEX5 export, as new receptor molecules can only enter the lumen once the previously imported ones have been retrieved. Notably, the proposed model does not contradict a possible role of PEX14 in recruiting cargo to peroxisomes (39).

The YG meshwork is suspended in the peroxisomal membrane by PEX13 molecules of opposite orientations. Such dual topology is unusual (56), and may be generated by random insertion of PEX13 directly into the peroxisomal membrane, likely with the involvement of PEX3. The dual topology facilitates assembly of the meshwork by allowing the YG domains to meet and associate within the plane of the membrane. The location of the YG meshwork directly in the lipid bilayer contrasts with the nucleoporin FG meshwork, which forms outside the nuclear membrane in the aqueous central channel of the nuclear pore complex (compare Figs. 8A and C). Our hydrogel experiments demonstrate that the N-terminal region preceding the YG domain can be accommodated in the YG meshwork. This observation explains why the N terminus of PEX13 can cross the membrane and become accessible to proteases and modification reagents, while the C terminus remains fixed in the membrane in one of the two orientations. Notably, the two orientations reconcile previously conflicting observations regarding the topology of the C terminus (39). The population of PEX13 with its PEX5-binding SH3 domain exposed to the cytosol could help recruit PEX5 to the organelle.

We suspect that the membrane-spanning walls encircling the YG meshwork are formed by the long AH of PEX13. The AH is the only feature of PEX13, besides the YG domain, that is strictly conserved. Our data show that the AH is also essential for import. Given its length (>60 residues) and dual topology, we surmise that alternating orientations of the AH assemble in a tilted manner into a pore-like structure in the peroxisomal membrane (fig. S11A), with their hydrophilic face oriented toward the aqueous YG phase in the middle and their hydrophobic face toward the lipids on the outside (fig. S11B). The diameter of this structure must be at least 9 nm (the size of the largest cargo reported to enter yeast peroxisomes) (19), but considerably smaller than the nuclear pore (~40 nm in yeast) (57). PEX13 has in fact been observed to form nanometer-sized clusters in the peroxisomal membrane by super-resolution microscopy (58), which are potentially consistent with such a conduit. By analogy to the nuclear pore, the concentration of YG domains inside the conduit must be very high (≥200 mg/ml). Our inability to reach such high concentrations in vitro likely explains why our YG hydrogels incompletely exclude inert material.

The similarity between peroxisomal import and nuclear transport raises the question of how proteins are targeted to the correct compartment. A possible answer is that NTRs have evolved to bind the phenylalanine-rich motifs of FG nucleoporins and exhibit low affinity for tyrosine (44). The peroxisomal meshwork might also be denser than the nuclear pore phase, given the shorter distance between aromatic residues in YG domains compared to nucleoporin FG domains. While peroxisomal import receptors bind the YG phase using unstructured segments (Fig. 8B), NTRs bind nucleoporin FG domains using large folded domains (59) (Fig. 8D). NTRs could thus be sterically impeded from penetrating the denser peroxisomal meshwork and would also have a lower affinity for the YG phase. PEX5 might not be impeded from traversing the nuclear pore, however, as it does leak into nuclei and maintains its steady-state nuclear exclusion only through continuous CRM1-mediated export (60). A similar export pathway does not exist in peroxisomes, which might therefore require a denser permeability barrier to exclude foreign proteins. Nevertheless, the barrier is likely not so tight as to exclude small molecules (35, 40). The limited permeability of the peroxisomal membrane might explain why a nuclear pore-like import mechanism, by which a folded protein crosses the membrane, can be accommodated. In contrast, other organelles (such as the ER and mitochondria) need a mechanism that maintains a tighter seal during protein translocation and thus require imported proteins to be unfolded.

## MATERIALS AND METHODS:

All reagents were obtained from Millipore-Sigma unless specified otherwise. Buffers were prepared in ultrapure water. RT denotes room temperature.

### Plasmid construction

The coding sequence of each recombinant protein was codon-optimized for *Escherichia coli* and inserted into plasmid pET-28b(+) by Gibson Assembly (from New England BioLabs). PEX5 proteins and cargo were produced as fusions containing N-terminal glutathione-S-transferase (GST). A 3C protease-cleavage site was introduced between the GST and the recombinant protein, preceded by the amino acid sequence GSD. The sequence coding for full-length, wild-type PEX5 corresponds to isoform X3 of the *Xenopus laevis* PEX5.S gene (GenBank accession no. XP_018082765.1). The fragment corresponding to the N-terminal region spans amino acids 1–268, whereas the fragment corresponding to the cargo-binding TPR domain spans amino acids 260–579. WxxxF/Y motifs in the N-terminal region were converted to AxxxA by mutagenesis. GFP fusions to PEX5 or to the fragments reported in the text were generated by inserting the sequence encoding mEGFP downstream of each protein, separated by either a GGSGGS linker (for full-length PEX5 or the TPR domain) or a GS linker (for the N-terminal region). GFP-SKL was assembled by fusing a modified peroxisomal targeting signal from *Photinus pyralis* (firefly) luciferase (i.e., the amino acid sequence YKGLKSKL) (47) to the C terminus of mEGFP, preceded by a glycine. GFP alone corresponds to mEGFP without the targeting signal. The plasmid encoding the N-terminal region of PEX5 fused to an anti-GFP nanobody was described previously (10).

Fragments encoding the YG domain of PEX13 were produced as fusions to an N-terminal 14× polyhistidine (14×His) tag corresponding to the amino acid sequence SKHHHHSGHHHTGHHHHSGSHHHTGS. A tobacco etch virus (TEV) protease-cleavage site was introduced between the 14×His tag and the recombinant protein. The fragment encompassing the entire unstructured N terminus of PEX13 corresponds to amino acids 1–193 of PEX13 from *Arabidopsis thaliana* (GenBank accession no. NP_187412.1); the YG domain alone corresponds to amino acids 76–193. Tyrosines within the YG domain were mutated to serines by mutagenesis. All constructs additionally included a single engineered cysteine at the C terminus for covalent derivatization.

Constructs designed for gene deletion and gene expression cassette integration in *Saccharomyces cerevisiae* (yeast) were assembled by restriction-enzyme cloning or Gibson Assembly. The coding sequence of yeast PEX13 (UniProt accession no. P80667) represents the wild type (WT). Conventional sequences for FLAG and SBP epitope tags, as well as cleavage sites for 3C and TEV proteases, were incorporated into PEX13 at the positions indicated in the text. Constructs containing a C-terminal TEV protease-cleavage site additionally included a diserine linker between the cleavage site and the PEX13 coding sequence. All cysteine point mutations reported in the text were made by mutagenesis, as well as mutations in the hydrophilic face (mut ζ: Q150A, E153A, Q164A, and E167A) or the hydrophobic face (mut φ: L155E and L166E) of the AH in PEX13. Conversion of tyrosines in the YG domain of PEX13 (amino acids 71, 81, 86, 90, 95, 101, 108, 110, 114, 115, 119, 123, 127, and 133) into either serines or phenylalanines was performed by *de novo* gene synthesis (by Integrated DNA Technologies).

To replace the YG domain (amino acids 70 to 134) of yeast PEX13, the sequences of YG domains from the following organisms were used: *Homo sapiens* (human; amino acids 67–121, UniProt accession no. Q92968); *Drosophila melanogaster* (fruit fly; amino acids 70–133, UniProt accession no. Q7JRD4); *Arabidopsis thaliana* (plant; amino acids 79–187, UniProt accession no. Q9SRR0); *Pichia Pastoris* (amino acids 25–114, UniProt accession no. C4R2I6); *Torulaspora delbrueckii* (amino acids 59–135, UniProt accession no. G8ZRC9); *Dictyostelium discoideum* (amoeba; amino acids 99–214, UniProt accession no. Q54CL3); and *Chlamydomonas reinhardtii* (algae; amino acids 70–194, UniProt accession no. A0A2K3CZW8).

All constructs were verified by sequencing.

### Yeast strains and culture conditions

All yeast strains reported in this study were derived from the *S. cerevisiae* parental strain UTL7A (*MAT*a, *ura3–52*, *trp1*, *leu2–3/112*). Strains were maintained on YPD medium (1 % w/v yeast extract, 2 % w/v peptone, 2 % w/v dextrose) with or without the antibiotics hygromycin (250 μg/ml; from Fisher, no. 10687010), geneticin (200 μg/ml; from Fisher, no. 10131035), or nourseothricin (100 μg/ml; from Jena Bioscience, no. AB-101), as appropriate. Genomic deletions and insertions were performed by homologous recombination using lithium acetate-based transformation (61).

The UTL7A strain was first derivatized by integrating the expression cassette for the violacein biosynthetic pathway (see below) at the *leu2* locus. Briefly, the plasmid pWCD1401 (35) was linearized by the restriction enzyme *Not*I, and the excised 11.5-kb fragment was purified by agarose gel electrophoresis and used to transform exponentially growing UTL7A cells. Clones were selected on synthetic defined (SD) medium containing 2 % w/v glucose and 6.7 g/L yeast nitrogen base without amino acids (from BD Difco), and supplemented with an amino acid mixture lacking leucine (from Sunrise Science). Correct integration of the cassette was confirmed by PCR, and the resulting violacein-positive (Vio^+^) strain was used to generate all subsequent strains and corresponds to the wild type.

All gene deletions utilized the *natMX* nourseothricin-resistance cassette as the selective marker. The cassette was amplified from plasmid pFA6A-*natMX* (from Addgene, no. 19343) by PCR, using primers that introduced 60-bp overhangs corresponding to the 5’ and 3’ untranslated regions immediately upstream and downstream of each gene’s open reading frame, respectively. Clones were selected on YPD medium containing nourseothricin (100 μg/ml), and replacement of each reading frame by the *natMX* cassette was confirmed by PCR.

To generate strains expressing mutant or epitope-tagged versions of the peroxisomal proteins described in the text, the *natMX* marker in the corresponding knockout strain was replaced with each mutant’s coding sequence, using either the *hygMX* hygromycin-resistance or the *kanMX* geneticin-resistance cassette as the selective marker. Briefly, the relevant coding sequence was PCR-amplified either from plasmid pFA6A-*hygMX* (from Addgene, no. 19342) or pFA6A-*kanMX* (from Addgene, no. 39296), together with the corresponding antibiotic resistance marker, using primers that introduced 60-bp overhangs as above. Clones were selected on YPD medium containing hygromycin (250 μg/ml) or geneticin (200 μg/ml), as appropriate, and correct insertion of the mutant reading frame was validated by PCR.

### Peroxisomal matrix protein import assay

A modified violacein biosynthetic pathway (35) was used to quantitatively measure peroxisomal matrix protein import activity in *S. cerevisiae* cells. Briefly, an expression cassette encoding three enzymes (VioA, VioB, and VioE), which together produce a green pigment, was integrated into the genome along with a leucine auxotrophic marker as described above. The first two enzymes (VioA and VioB) reside in the cytosol, whereas the third enzyme (VioE) contains a peroxisome targeting signal and is sequestered inside peroxisomes when import is functional. Only when import is compromised does VioE accumulate in the cytosol and the green pigment is made. Pigment production is inversely proportional to import efficiency (35).

To measure pigment production, strains were cultured overnight in YPD medium at 30 °C with shaking. The next morning, cultures were diluted 150-fold in 3 ml of freshly prepared SD medium lacking leucine, and cultured at 30 °C for a further 48 h. Cells were collected by centrifugation and resuspended in 300 μl of glacial acetic acid (from Fisher). The cell suspension was transferred to 1.5-ml microfuge tubes and incubated for 10 min at 95 °C, then mixed by inversion and incubated for a further 10 min. Debris were sedimented by centrifugation at 8,000 × *g* for 5 min at RT, and the resulting supernatants were transferred to a 96-well, round-bottom black-walled plate (from Corning, no. 3792). Fluorescence of each solution was measured with a Bio-Tek Synergy Neo2 microplate reader, using excitation and emission bands of 535 ± 5 nm and 585 ± 5 nm, respectively. To calculate relative import activity, the fluorescence reading from each strain was normalized to the average reading from wild-type cells (set to 100 %) and *pex13*Δ cells (set to 0 %) by linear interpolation.

### Disulfide-mediated crosslinking

Yeast cells expressing FLAG-tagged PEX13 with introduced cysteines were cultured overnight in YPD medium at 30 °C. Cells were collected by centrifugation, washed once with water, and resuspended in cold lysis buffer (20 mM HEPES•KOH pH 6.8 at RT, 150 mM potassium acetate, 5 mM magnesium acetate, 250 mM sorbitol, and 1 mM EDTA) supplemented with 1 mM phenylmethylsulfonyl fluoride (PMSF) and a protease inhibitor cocktail (from Bimake, no. B14001) according to the manufacturer’s instructions. The cell suspension was transferred to 2-ml screw-capped tubes (from Sarstedt, no. 726994005) on ice, mixed with 0.5-mm pre-chilled glass beads, and the cells lysed by bead-beating on a Biospec Products Mini-Beadbeater-16 (no. 607) for four 30-sec cycles with 2-min cooling on ice in-between. The cell lysate was clarified by low-speed centrifugation at 2,000 × *g* for 5 min at 4 °C to remove intact cells and cellular debris, and the resulting supernatant was re-centrifuged at 20,000 × *g* for 10 min at 4 °C to sediment heavy membranes including peroxisomes. The final pellet was gently resuspended in assay buffer (20 mM HEPES•NaOH pH 7.2, 50 mM NaCl, 250 mM sucrose, and 1 mM EDTA) by pipeting followed by mixing on a rotator at 4 °C for 15 min.

The homogenous membrane suspension was treated with or without the oxidizing agent 4,4′-dithiodipyridine (Aldrithiol-4; from Millipore-Sigma, no. 143057), at the concentrations indicated in the text, for 30 min at 30 °C with gentle agitation. Where indicated, 0.5 % w/v of n-dodecyl-β-D-maltoside (DDM; from Anatrace, no. D310) was added before the oxidant to solubilize the membranes. Unreacted cysteines were quenched with 10 mM *N*-ethylmaleimide (NEM) at 30 °C for 5 min. Membranes were then solubilized by shaking for 1 h at 4 °C with 0.5 % w/v DDM, and the solubilisate clarified by centrifugation at 20,000 × *g* for 10 min at 4 °C. PEX13 was immunoprecipitated from the resulting supernatant using anti-FLAG M2 agarose beads (no. A2220), and eluted by heating in Laemmli buffer as described below.

### Protease protection

Homogenous membrane suspensions from yeast cells expressing PEX13 with protease-cleavage sites (and FLAG or SBP epitope tags), were prepared as described above. Suspensions were mixed with 2 μM homemade 3C protease, 2 μM homemade TEV protease, or both proteases together, as indicated in the text, and incubated for 16 h at 4 °C. Where specified, 10 mM NEM was included in the reactions to pre-inactivate the proteases. All reactions were ultimately quenched with 10 mM NEM, and membranes solubilized with 0.5 % w/v DDM for 1 h at 4 °C with shaking. The solubilisate was clarified by centrifugation at 20,000 × *g* for 10 min at 4 °C, and PEX13 was then immunoprecipitated from the resulting supernatant using either anti-FLAG M2 agarose or streptavidin agarose beads (from ThermoFisher, no. 20353), as indicated in the text. Precipitated material was eluted with 0.4 mg/ml 3×FLAG peptide (from Bimake, no. B23112) or 4 mM biotin, as appropriate, in assay buffer containing 0.05 % w/v DDM, before being prepared for SDS-PAGE.

For the experiment shown in Figure 5B, the membrane suspension was first digested with 3C protease as described above, then treated with 200 μM Aldrithiol-4 for 30 min at 30 °C to promote disulfide bond formation. Reactions were quenched with NEM and processed for SDS-PAGE.

### PEGmal modification

Homogenous membrane suspensions from yeast cells expressing FLAG-tagged PEX13 with introduced cysteines were prepared as described above. Suspensions were incubated for 90 min at 4 °C and gentle agitation with 2 mM methoxypolyethylene glycol maleimide (PEGmal) of the following sizes (all from Millipore-Sigma), as specified in the text: 10-kD (no. 712469); 5-kD (no. 63187); 2-kD (no. JKA3124); and 0.8-kD (no. 712558). Where indicated, 0.5 % w/v DDM was added before PEGmal to solubilize the membranes. Unreacted PEGmal was quenched with 10 mM cysteine, and PEX13 was immunoprecipitated with anti-FLAG M2 agarose and prepared for SDS-PAGE as described above.

### Electrophoresis and immunoblotting

Unless specified otherwise, samples were heated in Laemmli buffer (from Bio-rad, no. 1610747) for 5 min at 95 °C, with or without 50 mM dithiothreitol (DTT; from GoldBio), and electrophoretically resolved under denaturing conditions on 4–20 % TGX precast polyacrylamide gels (from Bio-Rad). For Coomassie-blue staining, gels were first fixed in 50 % v/v methanol and 10 % v/v acetic acid for 30 min at RT, then incubated for a further 30 min in 50 % v/v methanol, 10 % v/v acetic acid, and 0.001 % w/v Coomassie Brilliant Blue R-250 (from BioRad), and finally destained overnight in 10 % v/v acetic acid before being washed into water. For immunoblotting, proteins were transferred overnight onto nitrocellulose membranes (from Bio-Rad, no. 1620112). Membranes were blotted in TBST buffer (20 mM Tris•NaOH pH 7.5, 150 mM NaCl, and 0.1 % v/v Tween 20) containing 3 % w/v non-fat milk solids (from Apex, no. 20241) using antibodies against the FLAG epitope (no. F7425) or the SBP tag (no. MAB10764), and fluorescently-labeled secondary antibodies IRDye 800CW or IRDye 680CW (from LI-COR Biosciences), as appropriate. Blots were imaged on a LI-COR Odyssey M imaging system.

### Determination of expression levels

To validate expression of FLAG-tagged PEX13 constructs reported in this study, membranes from the relevant strains were first solubilized in 0.5 % w/v DDM as described above, followed by immunoprecipitation using anti-FLAG M2 beads before processing for SDS-PAGE.

### Bioinformatic analysis

The amino acid composition of the YG domain of PEX13 versus nucleoporin FG domains was calculated by the ProtParam tool on the ExPASy server (62), using the sequences of PEX13, NUP62, and NUP98 homologs from the organisms shown in Table S1. The number of amino acids between consecutive aromatic residues (i.e., the spacer length) in YG domains versus nucleoporin FG domains was calculated using a custom script and the sequences of PEX13, NUP62, and NUP98 homologs from the organisms listed in Table S1. Because FG repeats consist not only of individual FG motifs but also of FxFG motifs (where “x” denotes any amino acid), such tandem motifs were considered as single aromatic clusters for calculating the spacer length.

### Protein purification

Recombinant proteins were produced in *E. coli* BL21 Rosetta 2(DE3) cells (from Novagen) by induction with isopropyl β-D-1-thiogalactopyranoside (IPTG; from GoldBio, no. I2481C50). All PEX5 proteins and cargo were expressed and purified by glutathione-affinity and size-exclusion chromatographies as described previously (10). Briefly, the proteins were eluted from the glutathione resin by proteolytic removal of their GST tag, gel-filtered into 40 mM HEPES•KOH pH 7.8 at RT, 100 mM KCl, 250 mM sucrose, 1 mM MgCl_2_, and 1 mM DTT, and concentrated to 100 μM before snap-freezing in single-use aliquots.

PEX13 fragments containing the YG domain were purified under denaturing conditions. Bacteria transformed with the desired plasmid were cultured in baffled flasks on an orbital shaker at 37 °C in 2×YT medium (from Fisher) containing 50 μg/ml kanamycin and 34 μg/ml chloramphenicol. On reaching an OD of 0.6, the cultures were cooled at RT for 30 min, then supplemented with 1 mM IPTG and incubated with shaking at 30 °C for an additional 6 h. Cells were collected by centrifugation at 4,000 × *g*, rinsed in phosphate-buffered saline, and frozen. Cell pellets were resuspended in freshly prepared lysis buffer (8 M urea, 100 mM sodium phosphate, 10 mM HEPES•NaOH, 10 mM imidazole, 5 mM DTT, and pH adjusted to 8.0 at RT just before use), incubated for 30 min at RT, and then homogenized by sonication. The cell lysate was clarified by centrifugation at 15,000 × *g* for 30 min at 20 °C, and the resulting supernatant incubated with nickel-charged nitriloacetic acid (Ni-NTA; from ThermoFisher) resin for 1 h at RT with agitation. Beads were washed with an excess of lysis buffer, and then with an excess of gelation buffer (2 M urea, 50 mM HEPES•NaOH, and 1 mM EDTA, pH adjusted to 8.0 at RT just before use) supplemented with 1 mM tris(2-carboxyethyl)phosphine (TCEP; from GoldBio). Bound protein was eluted with 500 mM imidazole in gelation buffer, supplemented with homemade 6×His-tagged TEV protease, and dialyzed overnight at RT against gelation buffer containing TCEP. The dialyzed solution was clarified by centrifugation, and passed over additional nickel resin to remove the released 14×His tag and TEV protease. The final flow-through served as the starting point for gelation.

### Preparation and photography of YG hydrogels

To initiate gelation, each recombinant fragment (dissolved in 2 M urea, 50 mM HEPES•NaOH pH 8.0 at RT, 1 mM TCEP, and 1 mM EDTA), was concentrated to 2 mM on a centrifugal filter device (from Amicon) at 40 °C. The resulting solutions were promptly injected into short pieces of Tygon S3 silicone tubing (from Saint-Gobain) that had been plugged at one end with hot-melt adhesive, and incubated at RT for several days to complete gelation. The contents of each piece of tubing were then squeezed out onto a colored grid, and photographed using a Canon EF 75–300 mm f/4–5.6 III zoom lens and a Canon EOS 20D digital single-lens reflex (DSLR) camera configured to maximize spatial resolution and minimize digital noise.

### YG-hydrogel permeation assay

A solution of the unstructured N-terminal region (including the YG domain) from *A. thaliana* PEX13 was concentrated to 2 mM in gelation buffer as described above. 2-μl drops were promptly spotted on the bottom of multiple wells of a 96-well glass-bottom plate (from Cellvis, no. P96–1.5H-N) and allowed to set for several hours at RT. The resulting gel droplets were equilibrated overnight in excess assay buffer (25 mM HEPES•KOH pH 7.8 at RT, 130 mM KCl), and imaged on a Leica SP8 X point-scanning confocal system, using a DMI6000 inverted microscope and a 20× 0.70 NA HC Plan Apochromat CS air objective. The objective was centered on the boundary between the buffer and gel, and focus maintained 5 μm above the glass surface by the Leica Adaptive Focus Control (AFC) system. Six initial frames were acquired at a rate of 2 frames per minute, and then a solution of fluorescently-labeled protein (as indicated in the text) in assay buffer was added and the acquisition continued for a further 30 min. All proteins were used at a final concentration of 500 nM. Images were acquired in Leica LAS X software using a pixel size of 1.14 μm^2^, scan speed of 100 Hz, and pinhole dilated to 1 Airy unit. Fluorescence was excited using a 490-nm bandlet selected from a white-light laser by an acousto-optic tunable filter (AOTF), and a 500–700-nm emission band was collected by a hybrid pixel detector (HyD) operating in standard mode without gating and gain set to 100. Imaging parameters were configured to maximize the signal-to-noise ratio (SNR) while avoiding saturation. All samples intended to be compared were imaged under identical acquisition settings.

### Image analysis

All analysis was performed in ImageJ (63) on the original, unmodified image data using routine functions. For images of immunoblots, band intensities were measured by densitometry. Fluorescence images of YG hydrogels were first background-subtracted and corrected for experimental variations in fluorophore concentration. To estimate the background, six successive images of the buffer–gel interface were acquired before addition of fluorescently-labeled protein, and then averaged. The resulting matrix was subtracted, pixel by pixel, from all frames of the timecourse acquired after addition of fluorescently-labeled protein. Background-subtracted timecourses were then normalized by mean fluorescence intensity in the buffer. To measure the protein concentration across the buffer–gel interface over time, a rectangular selection 60 × 300 μm was centered on the gel edge, and the mean fluorescence intensity across this field was measured at all timepoints using the plot profile function in ImageJ. The intensities were then normalized to the maximum value for each experimental cohort. To calculate the fold enrichment of each protein inside the gel, the mean intensity was measured inside a 15-μm arc that followed the inner contour of the gel edge at the 30-min timepoint. This value was then divided by the mean intensity in the buffer 150 μm away from the edge. To calculate permeation rates, the total area inside the gel occupied by the relevant permeating species at timepoint was identified by intensity-based thresholding. The resulting displacement values were plotted as a function of time, and fitted to straight lines whose slope corresponded to the permeation rate.

### Data plotting and statistical analysis

All experiments were independently performed at least three times. Data were plotted in GraphPad Prism (v. 9.3.1) and where indicated, statistical significance was calculated using Student’s two-tailed unpaired *t*-test. Fits to mathematical models were performed by nonlinear least squares regression in Prism.

### Image processing for publication and figure assembly

All images intended to be compared were processed identically. Fluorescence micrographs were first background-subtracted and corrected as described above, then linearly contrast stretched in ImageJ to the same bit range. Digitized images of immunoblots and stained gels were contrast stretched to reveal relevant bands but avoid clipping of the background. Digital photographs of YG hydrogels were processed using the Camera Raw plugin (v. 14.5.0.1177) in Adobe Photoshop (v. 23.5.1). Helical wheel diagrams were prepared using HeliQuest (64). Secondary structure predictions were performed with AlphaFold (65). Figures were assembled for publication in Adobe Illustrator (v. 25.4.1).

## Supplementary Material

Fig S1-S11 Table S1

## Data Availability

All data are available in the main text or the supplementary materials; the original unmodified image data are available from Mendeley Data (66) Reagents generated by this study are available from the corresponding author with a completed Materials Transfer Agreement.
