# Supplementary material for: Protein import into peroxisomes occurs through a nuclear pore-like phase
Source: Science. Author manuscript; Available in PMC 2022 Dec 28. (PMC9795577; doi:10.1126/science.adf3971)
Supplement: Fig S1-S11 Table S1 [file NIHMS1859017-supplement-Fig_S1-S11_Table_S1.docx]

­
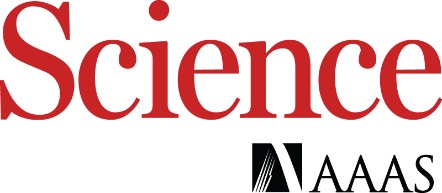


Supplementary Materials for

Protein import into peroxisomes occurs through a nuclear pore-like phase

Yuan Gao, Michael L. Skowyra, Peiqiang Feng, and Tom A. Rapoport

Correspondence to: [tom_rapoport@hms.harvard.edu](mailto:xxxxx@xxxx.xxx)

**This PDF file includes:**

Figs. S1 to S11

Table S1

**Fig. S1. Comparison of the YG domain of PEX13 to nucleoporin FG domains.** (**A**) Violin plot depicting the frequency of individual amino acids in the YG domain of PEX13 from organisms representative of different eukaryotic phyla (*n* = 16 organisms). The shape of the density curve for each amino acid represents all calculated values: the width is proportional to the density; horizontal lines designate the median; and the vertical spread of the curve spans the range. (**B**) Histogram showing the number of amino acids (i.e., the spacer length) between consecutive aromatic residues (tyrosine or phenylalanine) in the YG domain of PEX13 from the same organisms as in (A). Red line corresponds to the Gaussian frequency distribution fitted to the data. (**C** and **D**) As in (A) and (B), except calculated for the FG domains of nucleoporin NUP98 (*n* = 16) and NUP62 (*n* = 14) homologs. Because FG repeats consist not only of individual FG motifs but also of FxFG motifs (where "x" denotes any amino acid), such tandem motifs were considered as single aromatic clusters for calculating the spacer length.

**
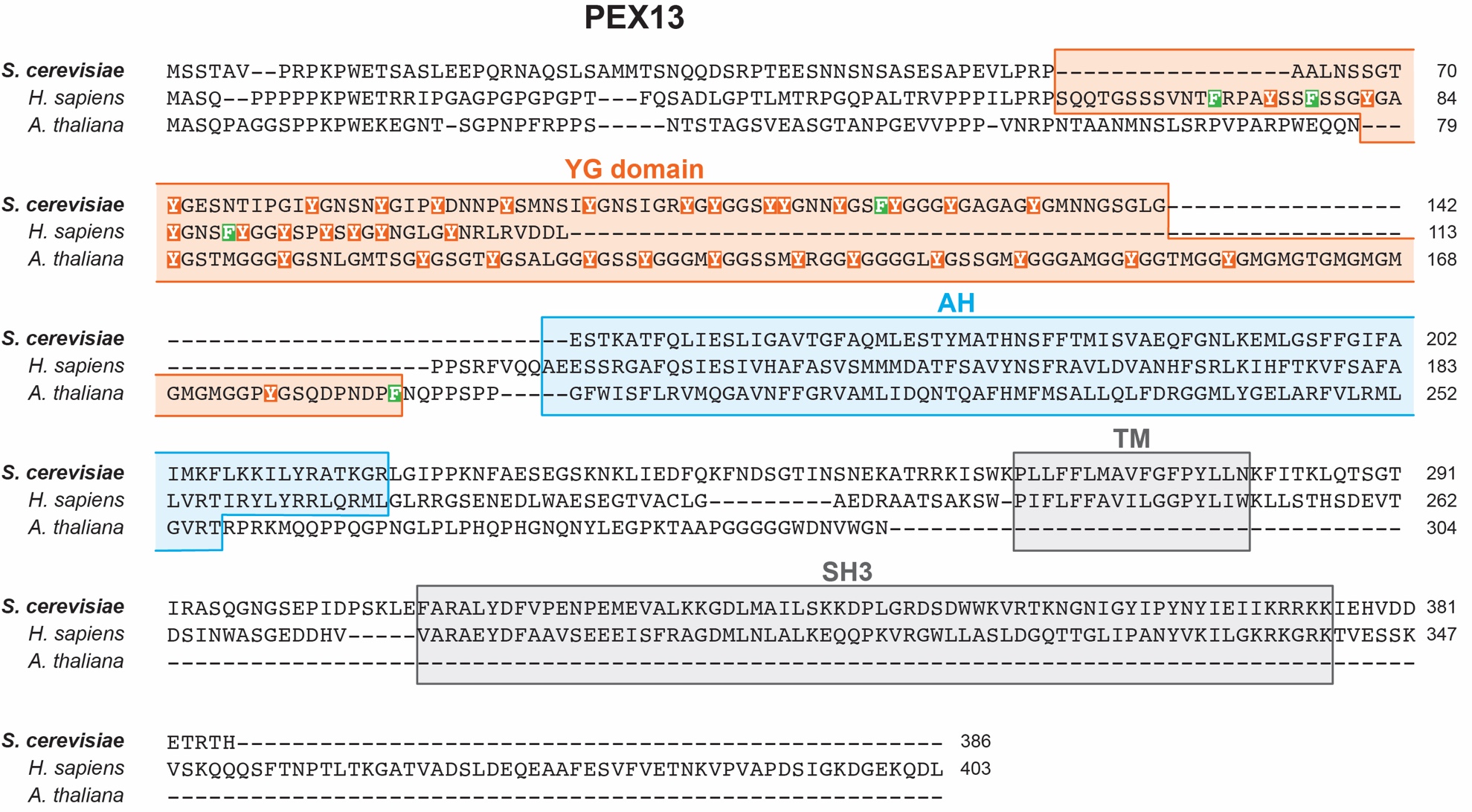
Fig. S2. Sequence alignment of PEX13 homologs.** Shown are the amino acid sequences of PEX13 from the indicated species, with the YG domain boxed in orange, the amphipathic helix (AH) in blue, and the transmembrane segment (TM) and SH3 domain in gray. Amino acid coordinates are indicated on the right of the align­ment.

**
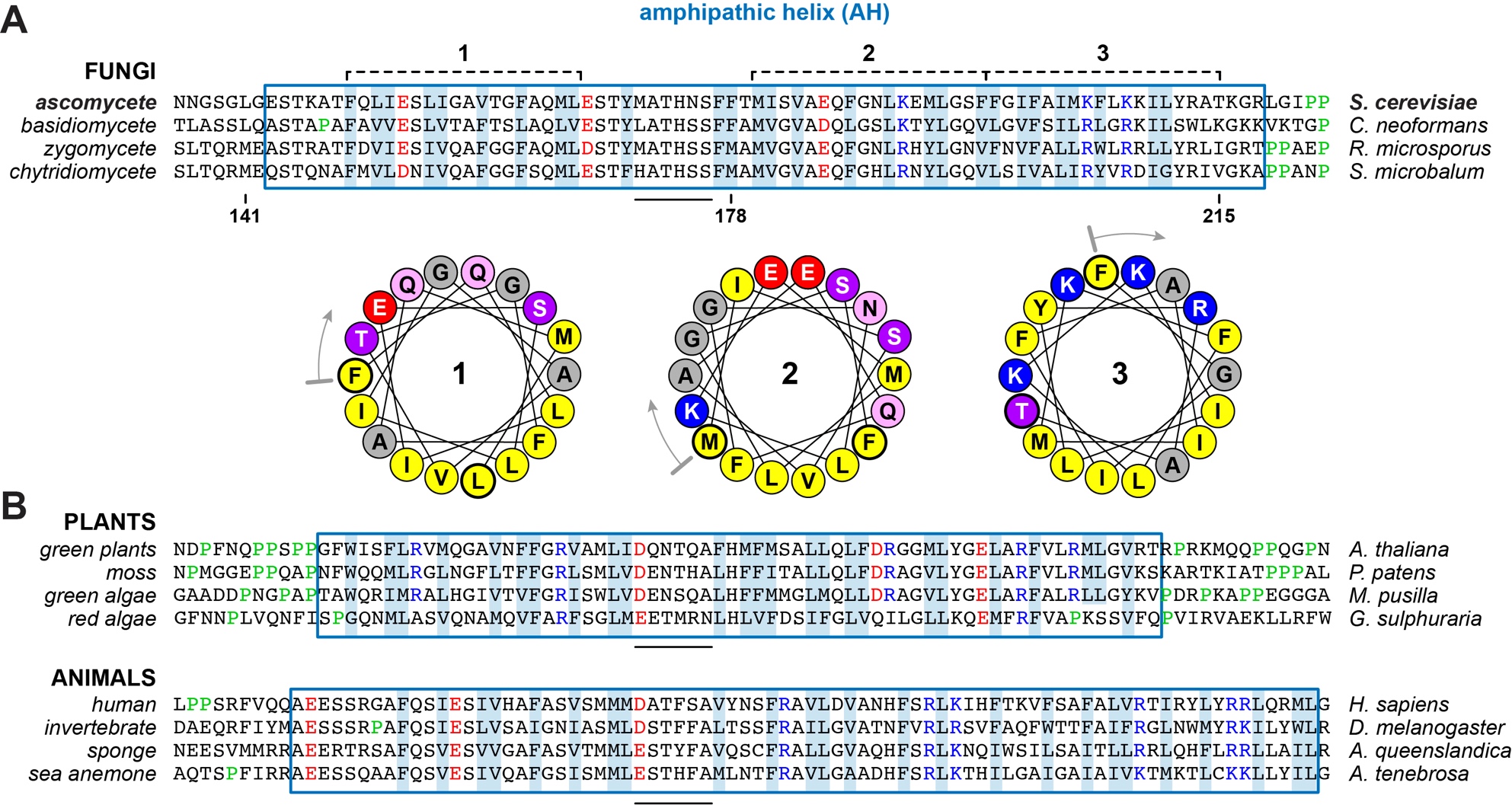
Fig. S3. Conservation of the amphipathic helix of PEX13.** (**A**) The alignment shows the amino acid sequence of the predicted amphipathic helix (boxed in blue) of PEX13 from organisms representative of different fungal phyla. Conserved hydrophobic residues are highlighted in light blue; amino acid positions beneath the alignment are given relative to the *S. cerevisiae* protein. The solid horizontal black line designates a short segment in the middle of the helix that lacks conserved hydrophobic residues. Helical wheel diagrams depict the asymmetric distribution of hydrophobic amino acids along one side of the helix of *S. cerevisiae* PEX13. The segments represented by the helical wheels are delineated by black dashed lines above the alignment; gray arrows indicate the N terminus of each segment and the direction of rotation. (**B**) As in (A), except showing sequence alignments of the predicted amphipathic helix from organisms representative of different plant and animal phyla.

**Fig. S4. Characterization of PEX13 in *S. cerevisiae* cells.** (**A**) Effect of tyrosine mutations in the YG domain of PEX13 on peroxisomal matrix protein import. Scheme shows the positions of all tyrosines (white lines) in the YG domain of yeast PEX13, which were mutated to serines or phenylalanines as indicated below. All constructs included a FLAG tag at their N terminus. Import activity of each mutant is plotted on the right, relative to wild-type (WT) PEX13 (mean ± standard error of 3 experiments). The FLAG blot below the plot confirms the expression of all constructs. (**B**) Expression of the FLAG-tagged PEX13 constructs analyzed in Fig. 1B. Each protein was immunoprecipitated using FLAG antibodies from the corresponding yeast strain and detected by immunoblotting. (**C**) As in (B), but for the constructs analyzed in Fig. 1C. (**D**) FLAG-tagged PEX13, bearing two cysteine residues at the indicated positions in the YG domain, was expressed in wild-type (WT) cells or in cells lacking the indicated peroxisomal matrix protein import components. Intact membranes from each strain were treated with 0, 50, or 200 µM Aldrithiol (oxidant), and PEX13 was then immunoprecipitated with FLAG antibodies and analyzed by SDS-PAGE with or without the reducing agent DTT­.

**Fig. S5. Peroxisomal matrix protein import activity in select *S. cerevisiae* strains.**

(**A**) Import activity (mean ± standard error of 3 experiments) in yeast strains containing various cysteine substitutions in PEX13 and/or lacking the indicated import components, plotted relative to wild-type (WT). Y→S and Y→F denote conversion of all tyrosines in the YG domain of PEX13 into serines or phenylalanines, respectively. (**B**) As in (A), but with strains used in protease protection experiments. 3C and TEV denote introduced cleavage sites for 3C protease or TEV protease, respectively. Import activity in the *pex5∆* strain expressing tagged PEX13 was reproducibly lower than in *pex13∆* cells, perhaps because PEX5 influences the enzymatic pathway used for the assay.


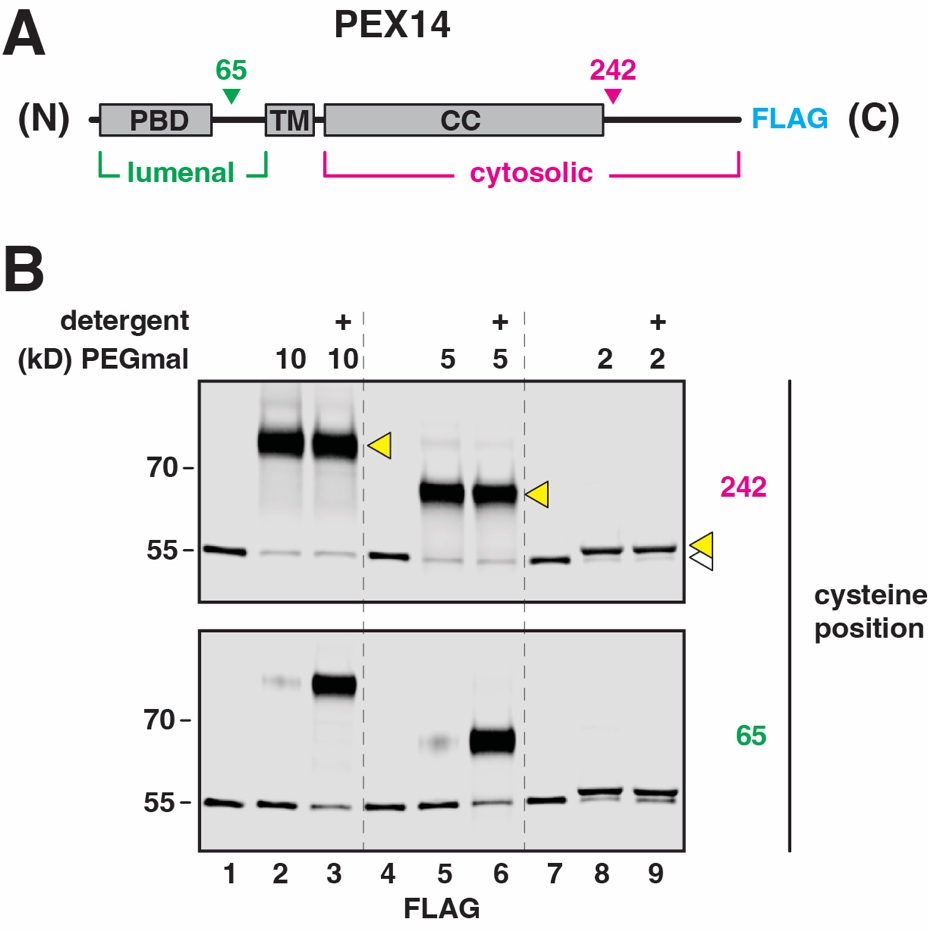


**Fig. S6. The peroxisomal membrane is permeable to small molecules.**

(**A**) To test the permeability of the peroxisomal membrane, individual cysteines were introduced into FLAG-tagged yeast PEX14 at the indicated positions. PEX14 is a single-pass transmembrane protein whose N terminus faces the peroxisomal lumen and C terminus faces the cytosol. Domains of PEX14 are designated as follows: PEX5-binding domain (PBD); transmembrane segment (TM); coiled-coil oligomerization domain (CC). (**B**) The resulting constructs were integrated into yeast, and intact membranes from the corresponding strains were treated with different sizes (in kD) of cysteine-reactive polyethylene glycol (PEGmal), in the presence or absence of detergent, then quenched with excess cysteine. Covalent modification of the proteins was visualized by immunoblotting for the FLAG tag. Modified and unmodified forms of the protein are designated by yellow and white triangles, respectively, in the upper blot. Note that the cysteine located in the cytosol was completely modified by all sizes of PEGmal in the absence of detergent, whereas the lumenal cysteine was only modified by the smallest PEGmal (2 kD).

**
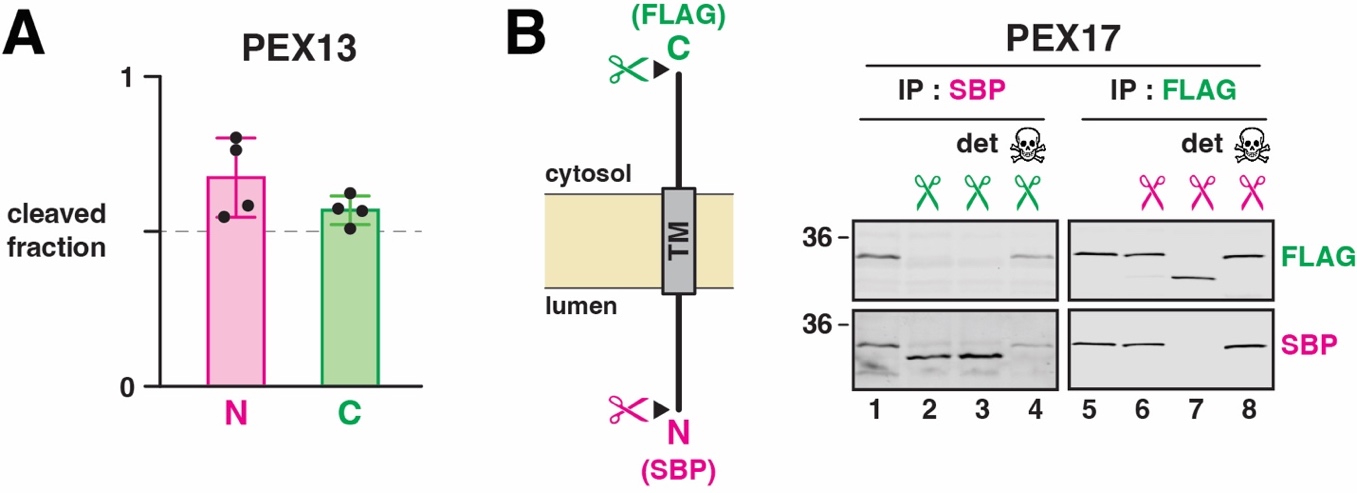
**

**Fig. S7. Membrane topologies of PEX13 and PEX17.**

(**A**) Fraction of total PEX13 that was proteolytically cleaved in the experiments shown in Fig. 3A (mean ± standard error of 4 experiments). N and C denote protease-cleavage sites at the N or C terminus of the protein, respectively. Note that the N terminus was sometimes more accessible than expected from an equal abundance of both orientations. (**B**) Scheme shows the location of 3C and TEV protease sites (scissors) and epitope tags that were introduced into yeast PEX17. Membranes containing the corresponding protein were treated with protease ± detergent (det), the reaction was then quenched with *N*-ethylmaleimide, and the protein immunoprecipitated (IP) as specified. Where indicated (crossbones), *N*-ethylmaleimide was added before the protease. The inferred orientation of PEX17 in the membrane is depicted in the scheme.

**
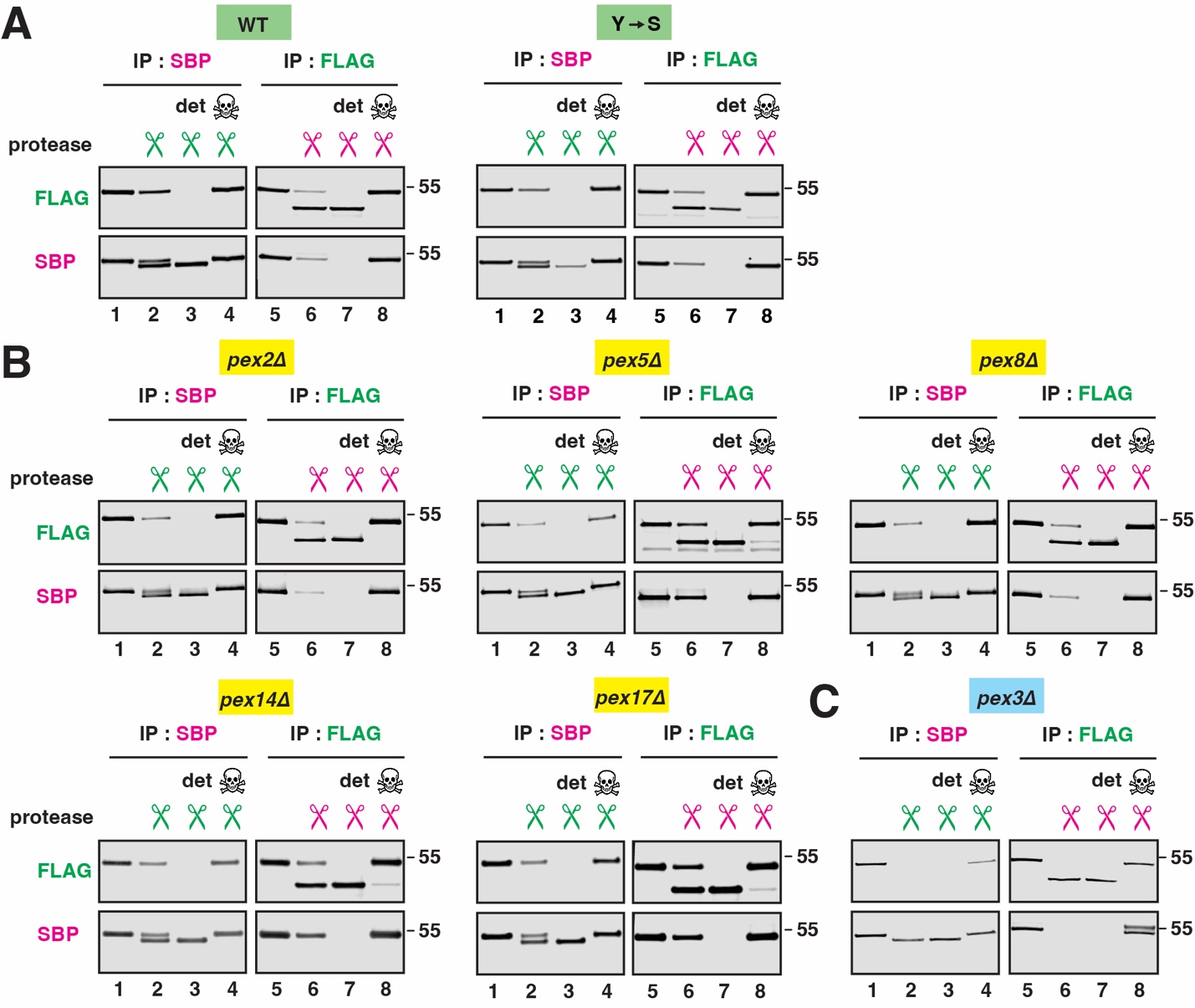
**

**Fig. S8. Membrane topology of PEX13.**

(**A**) As in Fig. 3A, except with constructs expressing wild-type (WT) PEX13 or a mutant in which all tyrosines of the YG domain were mutated to serines (Y→S). (**B**) As in (A), except with cells expressing wild-type PEX13 but lacking the indicated peroxisomal matrix protein import components. Note that the dual topology of PEX13 is unaffected in any of the strains. (**C**) As in (B), except with cells lacking the PEX3 component of the peroxisomal membrane protein insertion machinery. Note that PEX13 adopts only one membrane orientation in this strain.


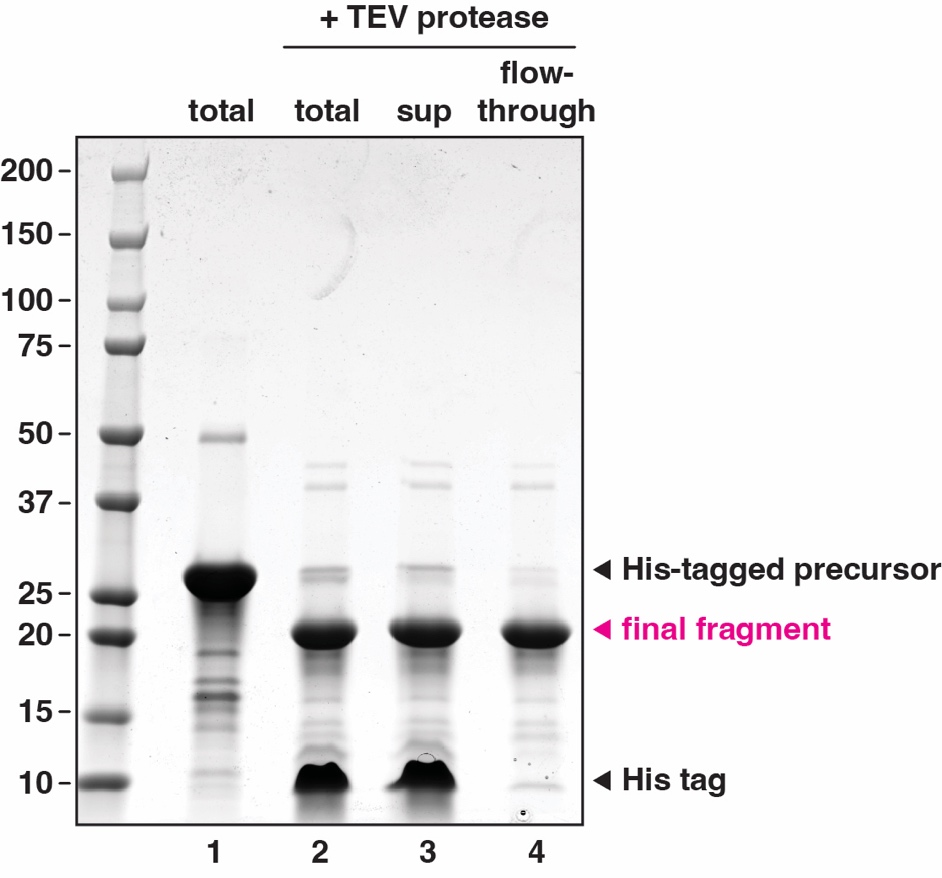


**Fig. S9. Purification of the recombinant YG-domain fragment from *A. thaliana* PEX13.**

Lane 1 shows the His-tagged precursor fragment purified by nickel-affinity chromatography in the presence of urea, as described in the Materials and Methods, stained by Coomassie Blue. Lane 2 shows the fragment after treatment with TEV protease to cleave off the His tag. Lane 3 shows the soluble fraction (sup) of the TEV-cleavage reaction, demonstrating that the fragment remains soluble in urea in the absence of the tag. The tag was removed by a subsequent nickel-affinity step, and the resulting flow-through containing the final purified fragment (magenta) is shown in lane 4. Relative molecular weights (in kD) are specified on the left.

**Fig. S10. Permeation properties of YG hydrogels.**

(**A**) The position of the permeation front (displacement) of the indicated constructs inside YG hydrogels over time, from Figure 5. Circles designate the average displacement from 3 experiments, and shaded areas span the range. Red lines are linear fits to the data, whose slope was used to calculate the permeation rate. (**B**) Mean permeation rates ± standard deviation of 3 experiments, calculated for the indicated constructs (ns, *p*-value = 0.32).


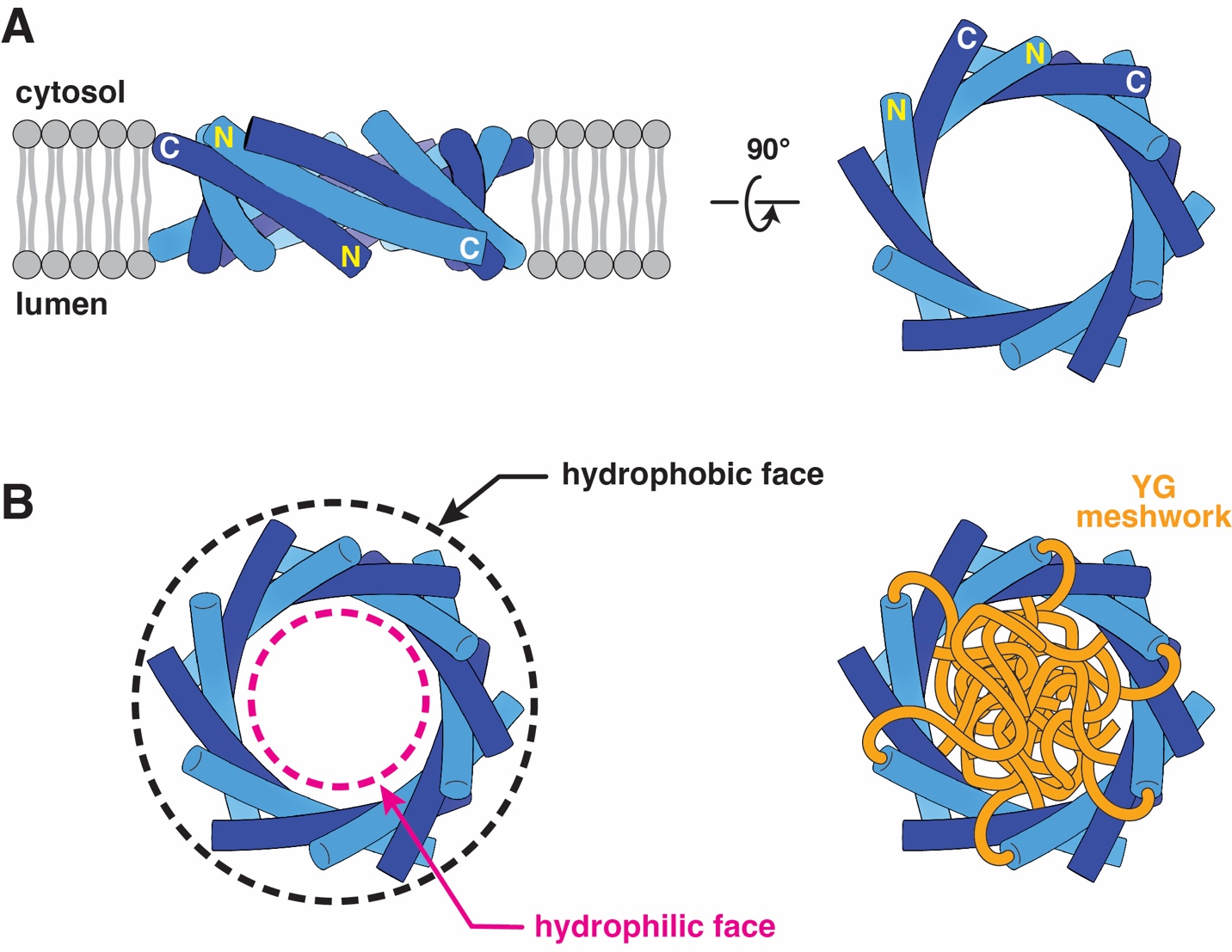


**Fig. S11. Model illustrating how the PEX13 amphipathic helix could form a membrane pore.** (**A**) Twelve copies of the PEX13 amphipathic helix were hypothetically arrayed around a circle in a tilted manner. Neighboring helices were arranged in alternating orientations (colored different shades of blue), as indicated by the positions of their N and C termini. Side and top views of the pore assembly are shown; the lipid bilayer is colored gray. (**B**) Scheme on the left depicts how the amphipathic helices have their hydrophobic surfaces (dashed black line) oriented toward the lipids outside the pore, and their hydrophilic surfaces (dashed magenta line) oriented toward the pore's interior. Scheme on the right illustrates how the YG domains from each subunit would form a dense meshwork inside the pore.

**Table S1. Accession numbers of PEX13, NUP62, and NUP98 homologs used for bioinformatic analysis.**

| **Organism** | | **PEX13** | **NUP62** | **NUP98** |
| --- | --- | --- | --- | --- |
| *S. cerevisiae* | ascomycete | P80667 | P14907 (NSP1) | P49687 (NUP145)  Q02629 (NUP100)  Q02630 (NUP116) |
| *Cryptococcus neoformans* | basidiomycete | Q5KKC0 | Q5KIE7 | A0A226A084 |
| *Batrachochytrium dendrobatidis* | chytridiomycete | F4PEP1 | F4NWJ7 | F4NVZ1 |
| *Rhizopus*  *microsporus* | zygomycete | A0A1X0S5S3 | A0A1X0RIK1 | A0A1X0SAQ0 |
| *A. thaliana* | land plant | Q9SRR0 | Q8L7F7 | Q8RY25 |
| *C. reinhardtii* | green algae | A0A2K3CZW8 | A0A2K3CV99 | A0A2K3E0Z7 |
| *Galdieria sulfuraria* | red algae | M2W636 | – | – |
| *Gracilariopsis chorda* | red algae | – | A0A2V3IPC1 | A0A2V3J4A8 |
| *H. sapiens* | vertebrate | Q92968 | P37198 | P52948 |
| *D. melanogaster* | invertebrate | Q7JRD4 | Q7JXF5 | Q9VCH5 |
| *Caenorhabditis elegans* | nematode | Q19951 | O01576 | G5EEH9 |
| *Oikopleura dioica* | tunicate | – | – | E4XLY7 |
| *Amphimedon queenslandica* | sponge | A0A1X7V2C0 | – | – |
| *Actinia tenebrosa* | sea anemone | A0A6P8H5J3 | A0A6P8I079 | A0A6P8JBW1 |
| *Monosiga brevicollis* | choanoflagellate | A9UX68 | A9UUV7 | A9VCQ3 |
| *Leishmania braziliensis* | euglenozoan | A4H9W4 | A4HKM6 | A4HFG3 |
| *D. discoideum* | amoebozoan | Q54CL3 | Q86IX4 | Q54EQ8 |
| *Tetrahymena thermophila* | ciliophoran | Q23KA1 | 01122680 (tetrathymena genome database) | – |
